# Integrative Analysis of the Mitochondrial Proteome in Yeast

**DOI:** 10.1371/journal.pbio.0020160

**Published:** 2004-06-15

**Authors:** Holger Prokisch, Curt Scharfe, David G Camp, Wenzhong Xiao, Lior David, Christophe Andreoli, Matthew E Monroe, Ronald J Moore, Marina A Gritsenko, Christian Kozany, Kim K Hixson, Heather M Mottaz, Hans Zischka, Marius Ueffing, Zelek S Herman, Ronald W Davis, Thomas Meitinger, Peter J Oefner, Richard D Smith, Lars M Steinmetz

**Affiliations:** **1**Institute of Human Genetics, GSF National Research Center for Environment and HealthNeuherbergGermany; **2**Institute of Human Genetics, Technical University of MunichMunichGermany; **3**Stanford Genome Technology Center and Department of Biochemistry, Stanford UniversityStanford, CaliforniaUnited States of America; **4**Environmental Molecular Sciences Laboratory, Pacific Northwest National LaboratoryRichland, WashingtonUnited States of America

## Abstract

In this study yeast mitochondria were used as a model system to apply, evaluate, and integrate different genomic approaches to define the proteins of an organelle. Liquid chromatography mass spectrometry applied to purified mitochondria identified 546 proteins. By expression analysis and comparison to other proteome studies, we demonstrate that the proteomic approach identifies primarily highly abundant proteins. By expanding our evaluation to other types of genomic approaches, including systematic deletion phenotype screening, expression profiling, subcellular localization studies, protein interaction analyses, and computational predictions, we show that an integration of approaches moves beyond the limitations of any single approach. We report the success of each approach by benchmarking it against a reference set of known mitochondrial proteins, and predict approximately 700 proteins associated with the mitochondrial organelle from the integration of 22 datasets. We show that a combination of complementary approaches like deletion phenotype screening and mass spectrometry can identify over 75% of the known mitochondrial proteome. These findings have implications for choosing optimal genome-wide approaches for the study of other cellular systems, including organelles and pathways in various species. Furthermore, our systematic identification of genes involved in mitochondrial function and biogenesis in yeast expands the candidate genes available for mapping Mendelian and complex mitochondrial disorders in humans.

## Abstract 2

Although individual approaches fall short, integrating multiple common genetic and biochemical approaches yields a description of mitochondrial proteins that is more than the sum of its parts

## Introduction

About half of the expected mitochondrial proteins in humans are known to date, and already a fifth of these known proteins are associated with human Mendelian disorders (Online Mendelian Inheritance in Man [http://www.ncbi.nlm.nih.gov/Omim/]; DiMauro and Schon 1998; Andreoli et al. 2004). Mitochondrial core functions such as oxidative phosphorylation, amino acid metabolism, fatty acid oxidation, and iron-sulfur cluster assembly have been highly conserved during evolution, suggesting that a systematic identification of mitochondrial proteins in model organisms will accelerate the search for new human mitochondrial disease genes (Steinmetz et al. 2002). In yeast, 477 proteins (469 encoded by the nuclear genome) show conclusive evidence of mitochondrial localization (this study and those listed in the Mitochondrial Proteome 2 [MitoP2] database [http://ihg.gsf.de/mitop]). About 30% of these proteins have evidence of orthologs in humans (MitoP2 database).

Identification of the yeast mitochondrial proteome is far from complete. Thirty to forty percent of the predicted complement of proteins that make up the organelle are still considered unknown although many genome-wide and functional systematic studies have been applied (Westermann and Neupert 2003). These include systematic identification of mitochondrial proteins by mRNA expression analysis under various conditions (DeRisi et al. 1997; Lascaris et al. 2003), DNA microarray analysis of mRNA populations associated with mitochondrion-bound polysomes (Marc et al. 2002), deletion phenotype screening (Dimmer et al. 2002; Steinmetz et al. 2002), large-scale localization studies (Kumar et al. 2002), protein–protein interaction studies (Uetz et al. 2000; Ito et al. 2001; Gavin et al. 2002; Ho et al. 2002), mass spectrometry (MS) of mitochondria (Pflieger et al. 2002; Ohlmeier et al. 2003), and various computational predictions of mitochondrial proteins (Nakai and Horton 1999; Drawid and Gerstein 2000; Small et al. 2004). In addition, two recent studies reduced the gap of missing mitochondrial localized proteins: a comprehensive proteomic study of mitochondria claimed to reduce the gap to 10% and identified 749 proteins (Sickmann et al. 2003), and a protein localization study identified 527 mitochondrial localized proteins by green fluorescent protein (GFP) tagging (Huh et al. 2003). Here we generated a component list of the mitochondrial organelle by first identifying mitochondrial proteins using MS and then integrating 22 datasets relevant to the study of mitochondria, including our proteomic data. The integration generated a comprehensive definition of the proteins involved in mitochondrial function and biogenesis and allowed for a comparison of genomic approaches, with implications beyond mitochondria.

## Results/Discussion

### Proteomics

We identified mitochondrial proteins by combining different methods for purification of whole mitochondrial organelles from yeast cell cultures and directly measured the proteins present in these fractions using MS. Mitochondria from yeast cells grown under four different conditions, including fermentable (glucose) and nonfermentable (lactate) substrates for both natural and synthetic culture media, were purified by either density gradient or free-flow electrophoresis. Preparations were separated into mitochondrial membrane and matrix fractions and analyzed separately for protein content. In total, 20 fractions were digested with trypsin and analyzed by reversed phase high resolution liquid chromatography/tandem MS (LC/MS/MS) (Ferguson and Smith 2003; Washburn et al. 2003). In addition, eight of the fractions were further analyzed by liquid chromatography/Fourier transform-ion cyclotron resonance MS (LC/FTICR) (Lipton et al. 2002; Smith et al. 2002). Altogether, 28 experimental datasets were generated (Table S1), which in combination identified 546 proteins (Table S2); listed also in the Yeast Deletion and Proteomics of Mitochondria [YDPM] database and the MitoP2 database).

**Figure 1:**
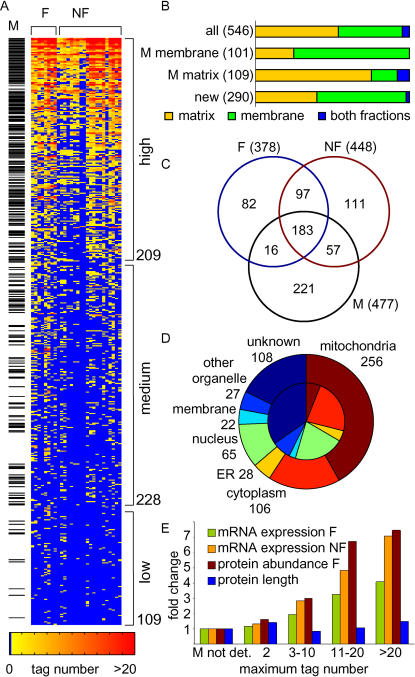
Enrichment for Mitochondrial Proteins by MS (A) shows the 546 proteins (in rows) identified from 28 datasets (columns). The proteins are sorted in decreasing order down rows by the number of experiments in which peptide tags were identified by MS and binned into three classes of detection frequency. The number at the bottom of each class indicates the total number of proteins in the class. Proteins that are part of the reference set, and thus are previously known mitochondrial proteins (M), are marked to the left. The experiments are divided according to fermentable (F) and nonfermentable (NF) mitochondrial preparations. (B) Proportions of proteins identified in membrane and matrix fractions. Whether a protein was detected predominantly in either the membrane or matrix fraction, or equal in both fractions, was determined based on where it was detected with an average higher tag number. Shown are the proportions for all 546 proteins, for known matrix proteins (i.e., matrix and intermembrane space, *n* = 109), for known membrane proteins (i.e., inner and outer membrane, *n* = 101), and for detected proteins not previously known to be mitochondrial (*n* = 290). (C) Distribution of proteins identified under fermentable and nonfermentable conditions by proteomics, and overlap with previously known mitochondrial proteins. Total numbers are given in parentheses. (D) Breakdown by localization of the 546 proteins identified. For mitochondrial localization the reference set was chosen; for localization outside mitochondria the GFP fusion protein data were used (Huh et al. 2003). The inner circle represents the distribution for all proteins in yeast. (E) Distribution of median mRNA expression under fermentable and nonfermentable conditions, protein abundance under fermentable conditions (Ghaemmaghami et al. 2003), and protein length across bins of confidence of identification (maximum number of tags identified in any of the 28 datasets). The bars indicate fold differences from the median for the known mitochondrial proteins that were not detected by MS (“M not det.”).

The performance of our proteomic and other systematic approaches in identifying mitochondrial proteins was evaluated against a reference set of 477 proteins classified as mitochondrial localized based on single gene studies. Of the 546 proteins identified by our proteomic approach, 47% were known mitochondrial, covering 54% of the reference set (256/477). Sorting the 546 candidates by the number of experiments in which they were found demonstrated that the probability of identifying a mitochondrial protein correlated with its detection frequency and with the confidence associated with its identification based on the number of peptide tags identified (Figure 1A). A separate analysis of membrane and matrix preparations showed that membrane and matrix proteins were more likely to be identified in membrane and matrix preparations, respectively. In addition, similar proportions of known mitochondrial proteins were identified from both fractions, indicating no significant bias towards the identification of either primarily soluble or primarily membrane-associated proteins (Figure 1B).

A comparison between fermentable and nonfermentable growth conditions revealed that more proteins were detected under respiration (448) than fermentation (378) conditions (Figure 1C), consistent with the known activation of oxidative phosphorylation during aerobic growth. Notably, of the 477 known mitochondrial proteins, 183 were identified under both growth conditions, suggesting that at least 38% of the mitochondrial machinery is present at moderate to high abundance even under fermentable growth conditions. This finding indicates the presence of a core mitochondrial protein set that exists under multiple growth conditions, which is consistent with previous observations (Ohlmeier et al. 2003).

Of the 546 proteins identified by proteomics, 182 proteins are known to localize outside mitochondria, mainly to the cytoplasm, nucleus, endoplasmic reticulum, and plasma membrane (Figure 1D). In addition to contaminants copurified with the fractions, identification of these proteins lends further support to the physical interaction of mitochondria with other cellular compartments and the existence of proteins with multiple localizations (Achleitner et al. 1999).

In the analysis of complex protein mixtures by MS, low abundance of proteins can preclude their identification (Patterson and Aebersold 2003). This might explain why 46% of the mitochondrial reference set escaped detection (221 proteins). To assess the correlation between protein detection and expression level systematically, we performed genome-wide mRNA expression analysis by means of high-density oligonucleotide arrays under the same fermentable and nonfermentable growth conditions. This analysis showed that absolute mRNA expression levels increased with the known index for establishing confidence of protein identification (tag number; Figure 1E): while genes identified by proteomics had median expression levels 1.2- to 7.1-fold higher than their unidentified mitochondrial counterparts, they did not differ in protein length, supporting a bias of current proteomic approaches primarily towards the detection of more abundant proteins.

**Figure 2:**
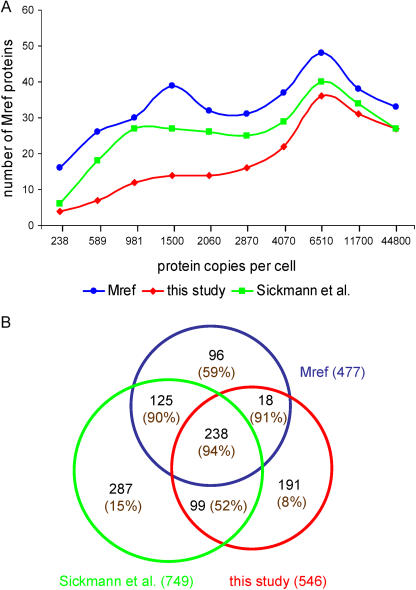
Evaluation of Proteomic Data for Protein Abundance and Mitochondrial Localization (A) Coverage of known mitochondrial proteins (Mref) by two MS proteome studies (this study and Sickmann et al. [2003]). We evaluated the 340 proteins of the mitochondrial reference set for which protein abundance data existed (Ghaemmaghami et al. 2003). The x-axis represents the median protein abundance of ten consecutive, equally sized bins of proteins. (B) Distribution and overlap of proteins identified by the two MS studies and known mitochondrial proteins. The total number of entries for each dataset is indicated in parentheses outside each circle. The number inside each circle indicates the number of proteins in each of the categories. In addition, the percentage of proteins that were localized to mitochondria by GFP tagging (Huh et al. 2003) is given in parentheses for each category.

We also extended our comparison to the analysis of protein abundance, which was recently determined for about two-thirds of the yeast proteome under fermentation (Ghaemmaghami et al. 2003). To visualize the distribution of identified proteins by their copy number per cell, we divided the 3558 proteins from that study into ten abundance classes, each consisting of an equal number of proteins. We then analyzed the distribution of known mitochondrial proteins across the classes. Figure 2A shows that we were able to detect known mitochondrial proteins over the whole range of expression levels, from 195 to 519,000 copies per cell. However, there is a clear bias towards the detection of more abundant proteins (i.e., in the highest abundancy class, 82% of the reference-set proteins were identified). A recently published study using multidimensional chromatography, Sickmann et al. (2003), achieved a higher coverage of known mitochondrial proteins, but the distribution of their identified proteins is also characterized by a bias against proteins of very low abundance (Figure 2A). Interestingly, even among the most abundant mitochondrial reference proteins, several remained undetected by either proteomic approach. Some of these proteins have a dual localization for which only a minor amount localizes to mitochondria (i.e., tRNA nucleotidyltransferase or synthases), further supporting the failure of proteomics to detect rare proteins in the samples.

Analysis of the overlap between both proteomic datasets (Figure 2B) shows that 337 proteins, corresponding to 62% of our study, were identified by both proteomic approaches, while 209 and 412 proteins, respectively, were present in only one or the other dataset. The majority of the proteins identified by both approaches were already known mitochondrial proteins (71%) or were localized to mitochondria by GFP-fusion proteins (an additional 13%; Huh et al. 2003). This high coverage stands in contrast to the much lower number found for proteins detected by only one dataset. Only 23% of the proteins identified by only one method were known mitochondrial proteins. In addition, while 52% of the new candidates (not previously known mitochondrial) identified by both proteomic studies were confirmed by GFP localization to mitochondria (Huh et al. 2003), only 8% and 15% of the candidates identified by only one or the other study were confirmed by the GFP-localization dataset. This analysis suggests that most of the proteins not found by both studies may be nonmitochondrial contaminants. Further indicative of this conclusion is the observation that proteins identified from localization categories outside mitochondria (see Figure 1D) also were among the high-abundancy proteins in those classes (data not shown). Since mitochondria were purified with different methods in the two proteomic studies, these observations suggest the importance of an integration of approaches.

### Integration

**Figure 3:**
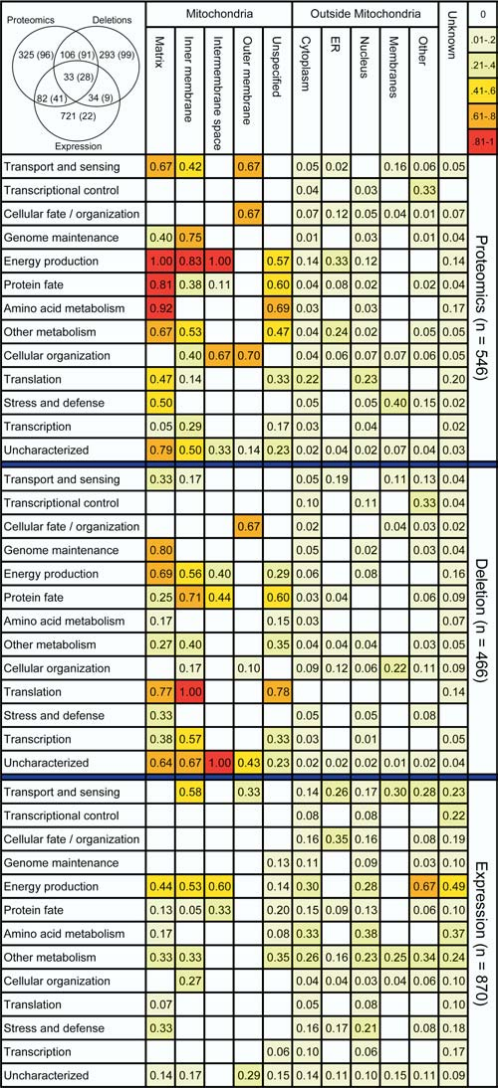
Functional Categories and Cellular Localization of Our Proteomic, Deletion, and Expression Datasets Each field shows the proportion of proteins found by the experiment out of the total number of proteins known with a given combination. Fields are color coded by the level of coverage gained by the experiment (color scale upper right). Localization outside mitochondria was based on the GFP fusion protein data (Huh et al. 2003). Fields with less than three identified proteins were not evaluated and left blank. In the upper left corner is the distribution and overlap of proteins identified by each experiment. In parentheses are the known mitochondrial proteins based on the reference set.

Are there classes of proteins that were not captured by a proteomic analysis, whether integrative or not, but that could be found using different approaches, and vice versa? To address this question, we performed a comparative analysis of functional categories identified by our proteomic dataset in comparison to functional approaches of gene expression analysis and quantitative deletion phenotype screening—datasets which were generated in this study and by Steinmetz et al. (2002), respectively. In the proteomic dataset, proteins annotated as localized outside mitochondria were not significantly enriched for any of the known functional classes (Mewes et al. 2002; Huh et al. 2003). In contrast, known mitochondrial proteins were primarily enriched for known mitochondrial functions such as energy production, transport and sensing, protein fate, and amino acid metabolism (Figure 3). Deletion phenotype screening enriched mainly for proteins involved in genome maintenance, transcription, and translation. Very low enrichment of mitochondrial proteins was achieved by mRNA expression, which predominantly detects proteins involved in energy production, the majority of which seem to localize outside the mitochondrial organelle.

A comparison of the distribution of protein enrichments shows that different functional categories are targeted by different approaches. Overall, the proteomic approach and the deletion approach identified about equal numbers of previously known mitochondrial proteins; however, they overlapped for less than 30% of the proteins. These observations suggest that combining complementary approaches and an integrative data analysis could be advantageous for predicting new mitochondrial proteins.

To improve our comparison of methods and to generate a high confidence list of mitochondrial proteins, we expanded our comparison to a total of 22 datasets relevant to the study of the mitochondrial proteome that had been collected to date. These included the experimental and computational approaches listed in the introduction, including a very recent proteome analysis (Sickmann et al. 2003) and GFP-tag localization study (Huh et al. 2003).

For most approaches the mitochondrial candidate genes were taken directly from the publication. From the mRNA expression analyses three datasets were generated. Genes were considered predictive of mitochondrial function if they were differentially expressed between fermentable and nonfermentable growth conditions (our study), differentially regulated in response to the diauxic shift (DeRisi et al. 1997), or differentially expressed in response to Hap4p overexpression (Hap4p is a transcription factor of mitochondrial proteins; Lascaris et al. 2003). The protein interaction datasets were screened for genes that interacted with known mitochondrial proteins. Most of the computational predictions searched for signal peptides indicative of mitochondrial targeting sequences. The homology studies searched for proteins similar to, for example, *Rickettsia prowazekii,* believed to be closest to a common ancestor with mitochondria. The details for each dataset are given in the MitoP2 database (a flatfile with the datasets is also available as Dataset S1).

**Figure 4:**
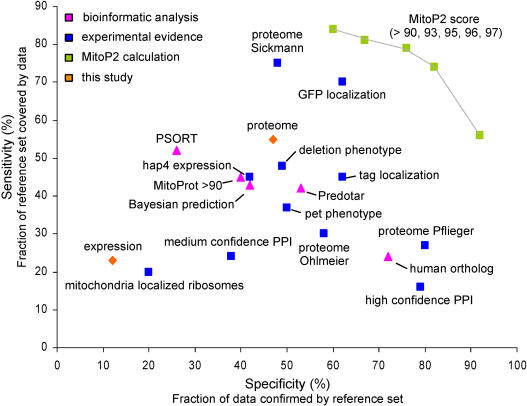
Specificity and Sensitivity of Systematic Approaches with Regard to Mitochondria Various datasets were benchmarked against the mitochondrial reference set. Each dot in the graph represents an entire dataset: PSORT (Nakai and Harton 1999), hap4 expression (Lascaris et al. 2003), deletion phenotype screen (Steinmetz et al. 2002), tag localization (Kumar et al. 2002), GFP localization (Huh et al. 2003), MitoProt greater than 90 (Scharfe et al. 2000), Bayesian prediction (Drawid and Gerstein 2000), pet phenotypes (Dimmer et al. 2002), three MS proteome studies (Pflieger et al. 2002; Sickmann et al. 2003; Ohlmeier et al. 2003), mitochondria localized ribosomes (Marc et al. 2002), Predotar (Small et al. 2004), and yeast proteins with known human mitochondrial orthologs (MitoP2 database). High-throughput protein–protein interaction datasets (PPI) were combined and divided into confidence classes (von Mering et al. 2002). Medium and high confidence PPI datasets were defined by interactions with known mitochondrial proteins (MitoP2 database). The predictive score for a mitochondrial protein (MitoP2) was based on the integration of 22 datasets, most of which are shown, and was calculated for different thresholds. Specificity and sensitivity are current best estimates owing to the incompleteness of the reference set.

We first assessed the performance of each method. For this purpose, the sensitivity and specificity of the different approaches were calculated by comparing each dataset with the reference set of known mitochondrial proteins (Figure 4). This comparison showed that the multidimensional proteomic data (Sickmann et al. 2003) covered 76% of the reference set (sensitivity of 76%), followed by the GFP fusion protein data (69%; Huh et al. 2003) and our proteomic dataset (54%). Among the experimental approaches that yielded sensitivity and specificity values of 45% or more were the same three datasets as above, in addition to the deletion phenotype screen (Steinmetz et al. 2002) and another localization study (Kumar et al. 2002). Fifty-three proteins were detected by all five methods, all of which were known mitochondrial proteins. In addition, only 51 proteins of the 477 mitochondrial reference-set proteins were not detected by any of these five methods. In comparison, a comprehensive dataset (union) of all 22 approaches covered 6,324 annotated open reading frames (ORFs) in which all 477 known mitochondrial proteins were included.

We next set out to determine whether the information supplied by each one of the different methods could be combined to achieve a predictive power that exceeded that of any single approach. We assessed the overlap among different combinations of the 22 datasets and defined a metric for attaching a numerical value to the likelihood of a protein being mitochondrial. A predictive score (MitoP2 score) was estimated based on the specificity of the best combination of approaches: we calculated for each approach as well as for all possible combinations of approaches, the percentage *(R)* of observed proteins present in the mitochondrial reference set relative to the total number of proteins detected. Most proteins belonged to more than one combination, and for these proteins multiple *R* values were calculated. The MitoP2 value was chosen to represent the highest *R* value calculated for a protein, representing the specificity of the best combination of methods.

Figure 4 shows that the list of proteins selected with the MitoP2 score yields a sensitivity and specificity higher than those achieved by any single approach. Among 435 proteins with a MitoP2 value greater than 96, 353 proteins were known mitochondrial. Using a MitoP2 value of 90 as a threshold, 691 yeast proteins were found of which 399 were known mitochondrial localized and 292 were new candidates. These data indicate that the power of defining mitochondrial proteins through combining various genome-wide datasets is significantly greater than that of any single method alone, including proteomics and GFP fusion protein localization.

**Figure 5:**
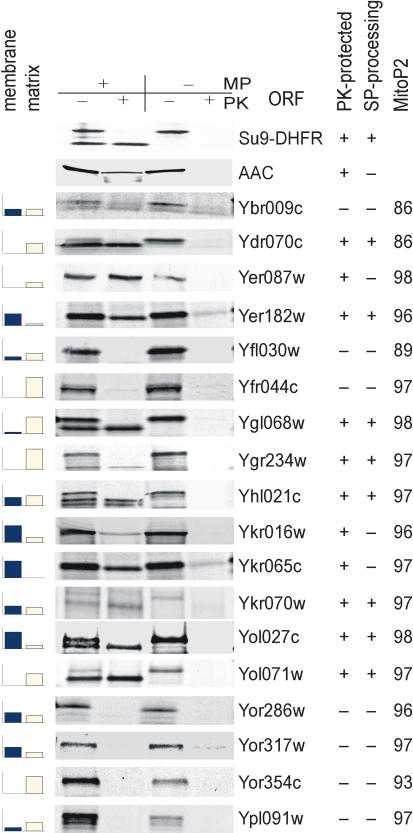
Verification of Proteomic Candidates by Mitochondrial Import Samples were incubated in the presence or absence of a membrane potential (MP) and of proteinase K (PK). Cases where import was accompanied by removal of the signal peptide (SP) are marked as “SP-processing” (+). Su9(1–69)DHFR and AAC serve as positive controls for a processed matrix protein and a nonprocessed inner membrane protein, respectively. The bar graphs indicate if a protein was more likely to be found in either the membrane or the matrix fractions of our proteomic data. The height of the bar corresponds to the number of samples in which a protein was identified with higher tag number—in the mitochondrial membrane or mitochondrial matrix fractions, respectively.

**Figure 6:**
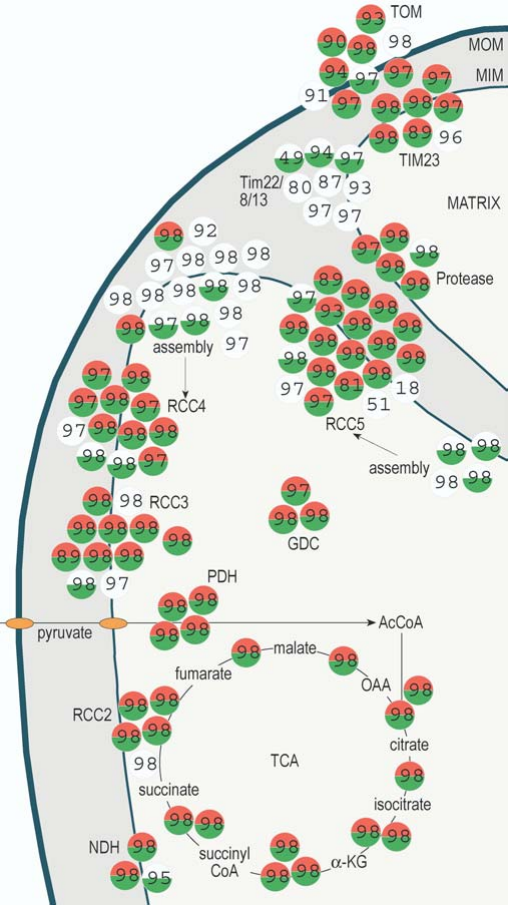
Verification of Prediction in Selected Mitochondrial Protein Complexes The assignment of complexes to mitochondrial compartments is based on known localizations of the protein subunits. Complexes are shown as clusters of circles, where each circle represents one protein. Red denotes a protein that was detected under fermentable and green under nonfermentable growth conditions by our proteomic dataset; white indicates proteins that were not detected. The numbers indicate the MitoP2 predictive score. For proteins without a number, no predictive score was assigned by the integrative analysis. Ac, acetyl; CoA, coenzyme A; α-KG, α-ketoglutarate; GDC, glycine decarboxylase; NDH, NADH-oxidoreductase; OAA, oxaloacetate; PDH, pyruvate dehydrogenase; RCC, respiratory chain complex; TIM, transport across inner membrane; TOM, transport across outer membrane; MOM and MIM, mitochondrial outer and inner membrane, respectively. A list of the genes for the plotted complexes is available in Table S4.

Three lines of evidence further support the success of this integrative analysis for defining the yeast mitochondrial proteome. First, the enrichment level for known mitochondrial proteins correlated with the level of the MitoP2 score and the number of experiments in which candidates were identified by proteomics: for the high, medium, and low classes (see Figure 1A) the median MitoP2 scores were 98, 94, and 82, respectively. Second, MitoP2 prediction was confirmed by import experiments. Ten out of 15 tested candidates with MitoP2 scores greater than 90 were imported into isolated yeast mitochondria, and seven of these were supported with signal sequence cleavage (Figure 5). This ratio (10/15) predicts that 67% of the 292 new candidates could be imported into mitochondria, indicating that 594 of the 691 proteins (with MitoP2 scores greater than 90) may thus be localized to mitochondria (399 plus 195). Third, an investigation of known subunits in mitochondria revealed that most of the components of known complexes were assigned a high MitoP2 score (Figure 6). Comparison with our proteomic dataset showed that while some of the assembly factors of respiratory chain complexes IV and V and subunits of the TIM22 complex were not detected by proteomics, the integrative analysis defined them correctly as mitochondrial proteins. This observation provides further support of the advantage gained by an integrative approach that combines various datasets.

### Implications

Our use of mitochondria as a model system for an integrative analysis of a subcellular proteome was aided by the large set of reference proteins known and previous experiments performed. All individual systematic approaches were biased to some extent and incomplete. An integration of data sources is therefore essential to go beyond the limitations of any single method and to achieve a more comprehensive view of the mitochondrial organelle. In similar approaches for other organelles and pathways, the use of reference sets to integrate functional genomic approaches and to define parts lists may prove useful.

Most of the mitochondrial reference proteins (399 of 477; 84%) had MitoP2 scores greater than 90, and since we have no evidence for a bias in the current reference set, the mitochondrial proteome as defined by the integration of 22 datasets is nearing saturation. In fact, our integration can be used to obtain an estimate of the number of mitochondrial proteins in yeast. Since outer membrane proteins are often not protease protected, the import analysis is conservative and allows us to estimate a lower boundary for the number of mitochondrial localized proteins. Considering that 84% of the reference proteins had MitoP2 scores greater than 90, we can predict a lower bound estimate of approximately 700 mitochondrial localized proteins in yeast (594 predicted true positives/0.84). This number is at the lower level of previous estimates and indicates that the mitochondrial organelle may consist of fewer proteins than the 800 anticipated (Westermann and Neupert 2003).

In order to make a prediction as to which combination of methods may be best applied to study a new system where no prior datasets exist, we performed an analysis of all pairwise combinations of methods. Among the comparisons, the union of proteomics (Sickmann et al. 2003) and subcellular localizations via GFP fusion proteins (Huh et al. 2003) achieved the highest coverage of previously known mitochondrial proteins (sensitivity 87%; specificity 45%). Higher specificity can be achieved by considering the overlap between the two datasets; however, coverage is then severely reduced due to a drastic reduction in gene number (sensitivity 58%; specificity 78%). Union of the two most complementary studies, our proteomics and deletion phenotype datasets—even though they are significantly less exhaustive—also achieved high values (sensitivity 76%; specificity 42%). If we concentrate on datasets that can be generated without massive genetic manipulations, as is required for gene tagging and deletion phenotype approaches, we can achieve a similar sensitivity of 78% with a specificity of 35% through combining in silico predictions (Predotar analysis; Small et al. 2004), expression profiling of a transcription factor mutant (Lascaris et al. 2003), and our proteomic data. These data argue that a combination of even a few complementary datasets may identify the majority of expected proteins. A better balance between sensitivity and specificity, however, can be achieved by an integrative analysis of as many complementary approaches as possible.

The advantage of integrative analysis combining structural and functional approaches is the high coverage of various mitochondrial components and functions. With this approach we were able to detect with high confidence proteins that had dual localization. For example, Met7p, which was assigned a MitoP2 score of 96, has a cytoplasmic and mitochondrial dual localization (DeSouza et al. 2000). Met7p was not detected as localized to mitochondria in any structural approach, but was identified by the deletion phenotype screen (Steinmetz et al. 2002). Altogether, 40 known mitochondrial reference proteins were not detected by proteomics or by subcellular localization studies. Through the inclusion of functional datasets in the calculations and the use of a localization list as a reference, our candidate list is strongly enriched for mitochondrial localized proteins, but is not limited to those. Consequently, because the MitoP2 calculation is based on both structural and functional datasets, the score not only predicts mitochondrial localized proteins but also reflects proteins that may localize outside mitochondria but affect mitochondrial function and biogenesis from there.

It is clear that the current list of mitochondrial proteins is not complete. The addition of further datasets will improve the prediction, as evidenced by the fact that less than 8% of known mitochondrial proteins have a MitoP2 score less than 70. These proteins thus remain rather undefined by the current integration, and further experimentation is needed to capture this class of mitochondrial proteins, consisting in part of three carrier proteins, 12 dual localized proteins, a few small proteins, and 11 mtDNA-encoded proteins (MitoP2 database). Our method of integration serves as one example; other ways of analyzing and integrating the datasets are possible and may reveal more proteins involved in other aspects of the mitochondrial system.

Finally, our study has implications for human diseases (Foury 1997). To date, 129 mitochondrial proteins have been implicated in human disorders (MitoP2 database; DiMauro and Schon 1998; Wallace 1999). The integration in yeast identified 143 new human orthologs of the 292 new yeast mitochondrial candidates defined by a MitoP2 score greater than 90 (Table S3 and MitoP2 database). This set of 143 proteins provides new candidates for putative human mitochondrial disorders where intervals have been mapped but no responsible gene has been identified to date (Steinmetz et al. 2002).

## Materials and Methods

### 

#### Purification of mitochondria

Saccharomyces cerevisiae strains were grown aerobically at 30 °C in SC or YP medium, and cells were harvested in logarithmic growth phase (OD600 < 1.3). Mitochondria were isolated by one of two different methods. One method involved differential centrifugation followed by a Nycodenz density gradient (Glick and Pon 1995), where the progress of mitochondrial purification was controlled by Western blot analysis using organelle-specific marker protein antibodies. In the other method, isolated mitochondria were purified by zone electrophoresis using a ProTeam FFE Free-Flow Electrophoresis apparatus (Tecan, Grödig, Austria) (Zischka et al. 2003). The anodic and cathodic circuit electrolytes consisted of 100 mM acetic acid and 100 mM triethanolamine acetate (pH 7.4). The electrolyte stabilizer was 280 mM sucrose, 100 mM acetic acid, and 100 mM triethanolamine (pH 7.4). The separation medium was 280 mM sucrose, 10 mM acetic acid, and 10 mM triethanolamine (pH 7.4). The counterflow medium was 280 mM sucrose. Table S1 lists the strains, growth conditions, and purification methods used for each dataset.

Prior to FFE fractionation, the mitochondria sample was equilibrated with separation medium and adjusted to a final protein concentration of 1–2 mg/mL. Electrophoresis was performed in horizontal mode at 5 °C with a total flow rate of 280 mL/h within the separation chamber at a voltage of 750 V. The samples were applied to the separation chamber with a flow rate of 1–2 mL/h via the cathodic inlet. Fractions were collected in 96-well plates, and the distribution of separated particles was monitored at a wavelength of 260 nm with a SynergyHT reader (Bio-Tek, Winooski, Vermont, United States). The peak fraction was isolated, shock-frozen in liquid nitrogen, and used for electron microscopy.

To assess purity, the preparations were analyzed by electron microscopy. The mitochondrial preparations were fixed with 4% formaldehyde, 2% glutaraldehyde, 4% sucrose, 2 mM calcium acetate, and 50 mM sodium cacodylate (pH 7.2) at 4 °C. The fixed samples were dissected with a scalpel, washed for 1 h in cacodylate buffer with 1% osmium tetroxide, and dehydrated with alcohol in increasing concentrations. After embedding in Araldite, the preparations were cut into 50-nm slices by means of an ultramicrotome (LKB-Produkter, Bromma, Sweden) and then analyzed on a Zeiss (Oberkochen, Germany) EM 10 electron microscope.

#### Fractionation of matrix and membrane proteins

Reagents used for the preparation of peptide samples were purchased from the indicated suppliers. Ammonium bicarbonate and methanol were from Fisher Scientific (Fair Lawn, New Jersey, United States). Sodium carbonate, urea, dithiothreitol, and calcium chloride were obtained from Sigma-Aldrich (St. Louis, Missouri, United States). Thiourea, trifluoroacetic acid, and acetonitrile were from Aldrich Chemical Company (Milwaukee, Wisconsin, United States). Sequencing-grade, modified porcine trypsin was obtained from Promega (Madison, Wisconsin, United States). Ammonium formate was obtained from Fluka (St. Louis, Missouri, United States). CHAPS and bicinchoninic acid (BCA) assay reagents and standards were from Pierce (Rockford, Illinois, United States). Purified water was generated using a Barnstead Nanopure Infinity water purification system (Dubuque, Iowa, United States).

Purified mitochondrial samples were disrupted using a Mini Beadbeater-8 (Biospec Products, Bartlesville, Oklahoma, United States) for 3 min at 4,500 rpm with 0.1 mm zirconia/silica beads (Biospec Products) in a 0.5-mL, sterile siliconized microcentrifuge tube. The lysed mitochondria, containing membrane and matrix proteins, were removed from the beads through a puncture at the bottom of the microcentrifuge tube, by centrifugation at 16,000 xg for 2 min at 4 °C, and the flow-through was collected in a second microcentrifuge tube. The collected lysate was then centrifuged at 356,000 xg for 10 min at 4 °C to pellet the mitochondrial membranes. The soluble supernatant was used for the study of mitochondrial matrix proteins, and the pellet was retained for identifying mitochondrial membrane proteins.

#### Mitochondrial membrane protein preparation

Using a sonication bath (Branson 1510, Danbury, Connecticut, United States), the membrane pellet was resuspended in 50 mM ammonium bicarbonate (pH 7.8) in an ice bath. The resuspended sample was diluted with ice-cold 100 mM sodium carbonate (pH 11.0) and incubated on ice for 10 min. The membranes were then pelleted by ultracentrifugation at 356,000 xg for 10 min at 4 °C. The pelleted membranes were washed using two aliquots of ice-cold water and pelleted again by centrifugation. The BCA protein assay was performed to determine protein concentration.

The membrane pellet was resuspended in 7 M urea, 2 M thiourea, 1% CHAPS in 50 mM ammonium bicarbonate (pH 7.8), using vortexing and sonication in an ice bath. Dithiothreitol was added to a final concentration of 9.7 mM in the resuspended sample, and the proteins were then treated with thermal denaturation for 45 min at 60 °C. The denatured and reduced protein sample was then diluted 10-fold with 50 mM ammonium bicarbonate (pH 7.8), and calcium chloride was added to a final sample concentration of 1 mM. Tryptic digestion was performed for 5 h at 37 °C using a 1:50 (w/w) trypsin-to-protein ratio. Snap-freezing the sample in liquid nitrogen quenched the digestion. The tryptic peptides were cleaned using a 1-mL strong cation exchange column (Discovery DSC-SCX , Supelco, Bellefonte, Pennsylvania, United States) per the manufacturer's instructions. The eluted peptide sample was concentrated by lyophilization and a BCA assay was performed to determine final peptide concentration. The peptide sample was stored at −80 °C until time for LC/MS/MS analysis.

#### Mitochondrial matrix protein preparation

The BCA protein assay was performed on the soluble matrix supernatant. The proteins were thermally denatured and reduced using 7 M urea, 2 M thiourea, and 5 mM dithiothreitol and incubating at 60°C for 30 min. The denatured and reduced protein sample was diluted 10-fold with 50 mM ammonium bicarbonate (pH 7.8), and the concentration of calcium chloride was adjusted to a final concentration of 1 mM. The tryptic digestion of the protein sample was performed in the same manner as described above for the membrane protein sample. The tryptic peptides were cleaned using a 1-mL LC-18 SPE column (Reversed Phase Supelclean LC-18 SPE, Supelco) per the manufacturer's instructions. The eluted peptide sample was concentrated by lyophilization, a BCA protein assay was performed, and the sample was stored at −80 °C until time for LC/MS/MS analysis.

#### Identification of potential mass and time tags by LC/MS/MS

The LC/MS/MS analysis of the tryptically digested peptides was performed as previously reported (Shen et al. 2001). In brief, the high-resolution reversed phase capillary liquid chromatography (LC) system was composed of a column assembled in-house using a 150-μm id × 360-μm od × 65-cm capillary (Polymicro Technologies, Phoenix, Arizona, United States) fixed with a 2-μm retaining mesh and packed with 3-μm Jupiter C18 stationary phase (Phenomenex, Torrence, California, United States). The column was equilibrated with 100% mobile phase A (0.05% trifluoroacetic acid in water) at 5,000 psi. Ten minutes after injecting a 10-μL sample (∼0.5 μg/μL), the exponential gradient began mixing mobile phase A with mobile phase B (0.1% trifluoroacetic acid:90% acetonitrile:9.9% water [vol/vol/vol]) while maintaining constant pressure. Using an in-house-manufactured electrospray ionization source, the capillary LC was interfaced with an LCQ ion trap mass spectrometer (ThermoFinnigan, San Jose, California, United States) with settings of 2.2 kV and 200 ^o^C for the ESI voltage and heated capillary, respectively. The data-dependent tandem MS analysis was conducted using a series of segmented mass/charge (*m/z*) ranges. A collision energy setting of 45% was employed for the collision-induced dissociation of the three most abundant ions detected in each MS scan. Dynamic exclusion was used to discriminate against previously analyzed ions. Peptides were identified by searching the tandem MS spectra against the complete annotated S. cerevisiae genome database (available at http://www.yeastgenome.org/) using SEQUEST (ThermoFinnigan) (Eng et al. 1994). “MudPIT” filtering rules were adopted as the acceptance criteria for peptides generated from the SEQUEST results (Washburn et al. 2001). Fully tryptic peptides with a 1+ charge state that had a cross-correlation (Xcorr) factor of 1.9 or greater were accepted. Fully or partially tryptic peptides with a 2+ charge state that had an Xcorr of 2.2 or greater were accepted as well. Peptides with a 2+ charge state that had an Xcorr of 3.0 or greater were accepted. Finally, fully or partially tryptic peptides with a 3+ charge state were accepted if an Xcorr of 3.75 or greater was obtained.

#### Identification of accurate mass and time tags by LC/FTICR

Some of the samples analyzed by LC/MS/MS were further analyzed by LC/FTICR. In LC/FTICR, tryptic peptides are analyzed using the same high-resolution reversed phase capillary LC described in the previous section, coupled to an electrospray ionization interface with a Fourier transform-ion cyclotron resonance mass spectrometer (Smith et al. 2002). We used both a custom-made 11.5 Tesla FTICR instrument, designed and constructed in house at Pacific Northwest National Laboratory, and a commercial 9.4 Tesla Bruker Apex III FTICR instrument (Bruker Daltonics, Billerica, Massachusetts, United States).

The acquired FTICR spectra (10^5^ resolution) were processed and deconvoluted using ICR-2LS (software written in-house at Pacific Northwest National Laboratory) to obtain peak lists containing the monoisotopic mass, observed charge, and intensity of the major ions in each spectrum. The masses were calibrated using the masses of internal calibrant peaks infused at the beginning and end of each LC/FTICR analysis. The peak lists for each analysis were then matched against the potential mass and time (PMT) tags defined previously (see above; by LC/MS/MS analyses among any of the previous samples) using VIPER (software written in-house at Pacific Northwest National Laboratory). The matching involved finding the groups of ions in the data, computing a median monoisotopic mass for each group, and then comparing the mass and elution time of the group with the mass and normalized elution time of each peptide in the PMT tag database (match tolerance of ± 8 ppm and ± 0.05 normalized elution time), resulting in the generation of an accurate mass and time (AMT) tag. Because the PMT tag database consisted only of the peptide tags produced via the previous LC/MS/MS analyses (a PMT tag database for the whole genome does not exist to date), the LC/FTICR analysis could identify only AMT tags which corresponded to previously identified PMT tags from one of the LC/MS/MS runs.

#### Identification of proteins

For the purpose of deriving a final list of proteins identified by MS, we included only proteins that had been detected by at least two tags in any single experimental dataset. As such we adapted the rules that are standard for minimizing false positives from MS and defining the detected proteins (Wu et al. 2003).

#### Gene expression profiling

Each sample was done in duplicate. Log phase cultures were grown overnight to an O.D. of 1 in 100 mL of YPD, YPL, SCD, or SCL medium. Total RNA was isolated using a hot phenol glass beads protocol. PolyA+ mRNA was purified using Qiagen's Oligotex kit (Qiagen, Valencia, California, United States). Then 4.5 μg of polyA+ mRNA were reverse transcribed to generate single stranded cDNA. Product was fragmented to approximately 50 bp using DNase digestion, biotin end labeled, and hybridized to Affymetrix S98 arrays as described in the Affymetrix user handbook (Affymetrix, Santa Clara, California, United States). Hybridizations were normalized and duplicate samples integrated to arrive at an estimate of absolute transcript abundance using the dChip computational package (Wong Lab, Harvard University). For genes with multiple probe sets on the array, only the probe set with the highest signal was used. For every gene, we calculated the fold difference between fermentable and nonfermentable growth conditions and considered significant only genes with a 1.2-fold or greater difference (either increased or decreased expression). In the final list we included only genes that showed a consistent direction of expression difference (increase or decrease) in both rich and synthetic media conditions.

#### Comparative genomic analysis between yeast and other organisms

All-against-all comparison of genes belonging to human, yeast, R. prowazekii, and Encephalitozoon cuniculi genomes has been conducted using the PSI-BLAST algorithm (Altschul et al. 1997). For each PSI-BLAST match, the following information has been stored in the MitoP2 database: the identification numbers of two matching proteins, the BLAST E-value of the match, the coverage of the BLAST alignment (defined as the fraction of amino acids of the shorter protein covered by the alignment), and whether the match is a bidirectional best hit (ortholog). A compendium of the yeast–human bidirectional blast hits for all yeast proteins with a MitoP2 score greater than 90 is given in Table S3.

#### Prediction of mitochondrial targeting sequences

Psort was downloaded locally as a perl5 script (from E-mail: nakai@imcb.osaka-u.ac.jp). MitoProt was run in the same way as in Scharfe et al. (2000). Predotar analysis was performed as described by Small et al. (2004). The protein lists are available in the MitoP2 database.

#### Integration of published datasets and calculation of MitoP2 score

To calculate the MitoP2 score, the percentage *R* of known mitochondrial proteins (reference set of 477 proteins) identified in each single genome-wide experiment (specificity) or in the overlap of all possible combinations of datasets (specificity of the combination of several methods) was calculated. Most proteins belonged to more than one combination, and for those proteins multiple *R* values were calculated. For example, proteins identified by two approaches received three *R* values: the specificity of the first approach alone, the specificity of the second approach alone, and the specificity of the overlap of both approaches. The MitoP2 value represented the highest *R* value calculated for a protein. The relevancy was checked according to the binomial law. The value gives a lower limit of the specificity of a defined combination because the mitochondrial reference dataset is not complete. For more detailed description, please see the MitoP2 database.

#### Protein import into isolated mitochondria

For T7 polymerase–driven synthesis of preproteins in vitro, the ORFs were amplified from ATG to STOP-codon by PCR, including the T7 RNA polymerase promoter and transcription initiation site within the 5′ primer. Using reticulocyte lysate (Promega), the resulting PCR products were utilized for coupled in vitro transcription/translation reactions to synthesize preproteins in the presence of 35S-radiolabeled methionine. Mitochondria were isolated by differential centrifugation from yeast strain W334 grown on lactate medium and resuspended at 25 °C in import buffer (0.3 mg/mL fatty-acid-free BSA, 0.6 M sorbitol, 80 mM KCl, 10 mM magnesium acetate, 2 mM KH2PO4, 2.5 mM EDTA, 2.5 mM MnCl2, 2 mM ATP, 5 mM NADH, and 50 mM HEPES/KOH [pH 7.2]). Import was initiated by adding 1% to 4% (vol/vol) of reticulocyte lysate containing radiolabelled preprotein. After 15 min, samples were placed on ice and subsequently treated with proteinase K (50 μg/mL) or not for 15 min to remove nonimported proteins. Protease was inhibited by the addition of 2 mM PMSF. Mitochondria were reisolated and analyzed by SDS-PAGE and autoradiography. Control experiments were performed in the absence of membrane potential in the presence of 1 μM valinomycin and 20 μM oligomycin.

## Supporting Information

Dataset S1 Flatfile with the Integrated Datasets (358 KB TXT).

Table S1 Sample Details for Each Proteomic Experiment (34 KB DOC).

Table S2 Proteins Identified by MS (544 KB DOC).

Table S3 Human Orthologs of Yeast Mitochondria-Related Proteins (828 KB DOC).

Table S4 Members of Selected Mitochondrial Protein Complexes (224 KB DOC).

### URLs

The YDPM database is the supporting online database for the proteomic, expression, and deletion datasets discussed in this paper, providing access to data analysis files, candidate lists, and a search function for individual ORFs. Available at http://www-deletion.stanford.edu/YDPM/YDPM_index.html.

The MitoP2 database is a mitochondrial proteome database for yeast and human that integrates published datasets and is available at http://ihg.gsf.de/mitop. The database provides annotated ORF information and the MitoP2 scores for the predicted mitochondrial proteins.

## Abbreviations

AMT: accurate mass and time
BCA: bicinchoninic acid
GFP: green fluorescent protein
LC: liquid chromatography
LC/FTICR: liquid chromatography/Fourier transform-ion cyclotron resonance mass spectrometry
LC/MS/MS: liquid chromatography/tandem mass spectrometry
MitoP2: Mitochondrial Proteome 2
MS: mass spectrometry
ORF: open reading frame
PMT: potential mass and time
Xcorr: cross-correlation
YDPM: Yeast Deletion and Proteomics of Mitochondria
