# Supplementary material for: Integrative Analysis of the Mitochondrial Proteome in Yeast
Source: PLoS Biol. 2004 Jun 15;2(6):e160. doi: 10.1371/journal.pbio.0020160 (PMC423137; doi:10.1371/journal.pbio.0020160)
Supplement: Table S1 — (34 KB DOC). [file pbio.0020160.st001.doc]

Table 1. Sample Details for Each Proteomic Experiment	
Sample	Media	Yeast Strain	Purification	YDPM File Nameb	
A	YP-Lactate	W303	DG	yplDG1_matY	
B	YP-Lactate	W303	DG	yplDG1_memY	
C	YP-Lactate	W303	DG	yplDG2_memY	
D	YP-Lactate	W303	DG	yplDG3_memY	
1	YP-Lactate	W303	FFE	ypl01_memY
ypl01_memY_FT
ypl01_matY
ypl01_matY_FT	
2	YP-Lactate	W303	FFE	ypl02_memY
ypl02_memY_FT
ypl02_matY
ypl02_matY_FT	
4	YP-Dextrose	W303	FFE	ypd04_memY
ypd04_matY	
5, 10, 11a	SC-Lactate	S1001	FFE	scl01_memY
scl01_memY_FT
scl01_matY
scl01_matY_FT	
7, 12a	SC-Dextrose	S1001	FFE	scd01_memY
scd01_memY_FT
scd01_matY
scd01_matY_FT	
13	YP-Dextrose	W303	FFE	ypd13_memY
ypd13_matY	
14	YP-Lactate	W303	FFE	ypl14_memY
ypl14_matY	
15	YP-Lactate	W303	FFE	ypl15_memY
ypl15_matY	

aIn rows with multiple samples, more than one culture was grown under the same conditions (same media, same strain), but mitochondria were purified separately. Due to low protein yield, corresponding fractions were joined for MS analysis.
bDatasets produced by MS: filenames ending with FT were derived from FTICR analysis, all others from LC/MS/MS. Filenames containing “mem” designate analysis of the mitochondrial membrane fraction; “mat,” of the mitochondrial matrix fraction. The datasets and more information about them are available at http://www-deletion.stanford.edu/YDPM/YDPM_index.html.
YP, yeast peptone; SC, synthetic complete; DG, differential centrifugation by Nycodenz density gradient; FFE, free-flow electrophoresis.
DOI: 10.1371/journal.pbio.0020160.t001
