# Supplementary material for: Integrative Analysis of the Mitochondrial Proteome in Yeast
Source: PLoS Biol. 2004 Jun 15;2(6):e160. doi: 10.1371/journal.pbio.0020160 (PMC423137; doi:10.1371/journal.pbio.0020160)
Supplement: Table S2 — (544 KB DOC). [file pbio.0020160.st002.doc]

**Table S1.** Proteins identified by mass spectrometry.

| ORF | GENE | Description (CYGD) |
| --- | --- | --- |
| *Q0045* | *COX1* | cytochrome-c oxidase subunit I |
| *Q0085* | *ATP6* | F1F0-ATPase complex, FO A subunit |
| *Q0115* | *BI3* | mRNA maturase BI3 |
| *Q0140* | *VAR1* | mitochondrial ribosomal protein |
| *Q0250* | *COX2* | cytochrome-c oxidase subunit II |
| *YAL001C* | *TFC3* | TFIIIC (transcription initiation factor) subunit, 138 kD |
| *YAL011W* | *SWC1* | weak similarity to Mus musculus p53-associated cellular protein |
| *YAL023C* | *PMT2* | mannosyltransferase |
| *YAL044C* | *GCV3* | glycine decarboxylase, subunit H |
| *YBL002W* | *HTB2* | histone H2B.2 |
| *YBL003C* | *HTA2* | histone H2A.2 |
| *YBL004W* | *UTP20* | weak similarity to Papaya ringspot virus polyprotein |
| *YBL005W-B* | *YBL005W-B* | TY1B protein |
| *YBL015W* | *ACH1* | acetyl-CoA hydrolase |
| *YBL022C* | *PIM1* | ATP-dependent protease, mitochondrial |
| *YBL027W* | *RPL19B* | 60S large subunit ribosomal protein L19.e |
| *YBL030C* | *PET9* | ADP/ATP carrier protein (MCF) |
| *YBL045C* | *COR1* | ubiquinol--cytochrome-c reductase 44K core protein |
| *YBL064C* | *PRX1* | strong similarity to thiol-specific antioxidant enzyme |
| *YBL072C* | *RPS8A* | ribosomal protein S8.e |
| *YBL079W* | *NUP170* | nuclear pore protein |
| *YBL087C* | *RPL23A* | 60S large subunit ribosomal protein L23.e |
| *YBL088C* | *TEL1* | telomere length control protein |
| *YBL092W* | *RPL32* | 60S large subunit ribosomal protein L32.e |
| *YBL095W* | *YBL095W* | similarity to C.albicans hypothetical protein |
| *YBL099W* | *ATP1* | F1F0-ATPase complex, F1 alpha subunit |
| *YBL101C* | *ECM21* | involved in cell wall biogenesis and architecture |
| *YBR003W* | *COQ1* | hexaprenyl pyrophosphate synthetase precursor |
| *YBR009C* | *HHF1* | histone H4 |
| *YBR010W* | *HHT1* | histone H3 |
| *YBR016W* | *YBR016W* | strong similarity to hypothetical proteins YDL012c and YDR210w |
| *YBR026C* | *ETR1* | mitochondrial respiratory function protein |
| *YBR031W* | *RPL4A* | ribosomal protein |
| *YBR037C* | *SCO1* | involved in stabilization of Cox1p and Cox2p |
| *YBR039W* | *ATP3* | F1F0-ATPase complex, F1 gamma subunit |
| *YBR048W* | *RPS11B* | ribosomal protein S11.e.B |
| *YBR054W* | *YRO2* | strong similarity to HSP30 heat shock protein Yro1p |
| *YBR058C* | *UBP14* | ubiquitin specific protease |
| *YBR063C* | *YBR063C* | hypothetical protein |
| *YBR084C-A* | *RPL19A* | 60S large subunit ribosomal protein L19.e |
| *YBR084W* | *MIS1* | C1-tetrahydrofolate synthase precursor, mitochondrial |
| *YBR085W* | *AAC3* | ADP/ATP carrier protein (MCF) |
| *YBR106W* | *PHO88* | involved in phosphate transport |
| *YBR118W* | *TEF2* | translation elongation factor eEF1 alpha-A chain, cytosolic |
| *YBR120C* | *CBP6* | apo-cytochrome B pre-mRNA processing protein |
| *YBR146W* | *MRPS9* | ribosomal protein S9 precursor, mitochondrial |
| *YBR176W* | *ECM31* | involved in cell wall biogenesis and architecture |
| *YBR177C* | *EHT1* | alcohol acyl transferase |
| *YBR179C* | *FZO1* | required for biogenesis of mitochondria |
| *YBR181C* | *RPS6B* | ribosomal protein S6.e |
| *YBR192W* | *RIM2* | mitochondrial carrier protein (MCF) |
| *YBR221C* | *PDB1* | pyruvate dehydrogenase (lipoamide) beta chain precursor |
| *YBR227C* | *MCX1* | similarity to E.coli ATP-binding protein clpX |
| *YBR230C* | *YBR230C* | hypothetical protein |
| *YBR262C* | *YBR262C* | questionable ORF |
| *YBR263W* | *SHM1* | serine hydroxymethyltransferase precursor, mitochondrial |
| *YBR269C* | *YBR269C* | weak similarity to 'cpa', phospholipase C, Clostridium perfringens |
| *YBR291C* | *CTP1* | citrate transport protein, mitochondrial (MCF) |
| *YCL009C* | *ILV6* | acetolactate synthase, regulatory subunit |
| *YCL017C* | *NFS1* | regulates Iron-Sulfur cluster proteins, cellular Iron uptake, andIron distribution |
| *YCL043C* | *PDI1* | protein disulfide-isomerase precursor |
| *YCL044C* | *YCL044C* | similarity to hypothetical protein K. lactis |
| *YCL057C-A* | *YCL057C-A* | strong similarity to hypothetical protein S.pombe |
| *YCL057W* | *PRD1* | proteinase yscD |
| *YCL064C* | *CHA1* | L-serine/L-threonine deaminase |
| *YCR005C* | *CIT2* | citrate (si)-synthase, peroxisomal |
| *YCR012W* | *PGK1* | phosphoglycerate kinase |
| *YCR028C-A* | *RIM1* | ssDNA-binding protein, mitochondrial |
| *YCR031C* | *RPS14A* | 40S Ribosomal protein S14.e |
| *YCR033W* | *SNT1* | similarity to nuclear receptor co-repressor N-Cor |
| *YCR073C* | *SSK22* | MAP kinase kinase kinase |
| *YDL004W* | *ATP16* | F1F0-ATPase complex, F1 delta subunit |
| *YDL015C* | *TSC13* | required for elongation of the very long chain fatty acid (VLCFA) moiety of sphingolipids |
| *YDL028C* | *MPS1* | serine/threonine/tyrosine protein kinase |
| *YDL058W* | *USO1* | intracellular protein transport protein |
| *YDL066W* | *IDP1* | isocitrate dehydrogenase (NADP+), mitochondrial |
| *YDL067C* | *COX9* | cytochrome-c oxidase chain VIIA |
| *YDL069C* | *CBS1* | translational activator of cob mRNA |
| *YDL081C* | *RPP1A* | 60S large subunit acidic ribosomal protein a1 |
| *YDL083C* | *RPS16B* | ribosomal protein S16.e |
| *YDL085W* | *NDE2* | mitochondrial NADH dehydrogenase that catalyzes the oxidation of cytosolic NADH |
| *YDL095W* | *PMT1* | mannosyltransferase |
| *YDL120W* | *YFH1* | regulates mitochondrial iron accumulation |
| *YDL125C* | *HNT1* | similarity to protein kinase C inhibitor-I |
| *YDL130W-A* | *STF1* | ATPase stabilizing factor, 10 kDa |
| *YDL133W* | *YDL133W* | hypothetical protein |
| *YDL136W* | *RPL35B* | 60S large subunit ribosomal protein |
| *YDL171C* | *GLT1* | glutamate synthase (NAPDPH) (GOGAT) |
| *YDL174C* | *DLD1* | D-lactate ferricytochrome C oxidoreductase (D-LCR) |
| *YDL178W* | *AIP2* | actin interacting protein 2 |
| *YDL181W* | *INH1* | inhibitor of mitochondrial ATPase |
| *YDL185W* | *TFP1* | H+-ATPase V1 domain 69 KD catalytic subunit, vacuolar |
| *YDL191W* | *RPL35A* | 60S large subunit ribosomal protein |
| *YDL198C* | *YHM1* | member of the mitochondrial carrier family (MCF) |
| *YDL202W* | *MRPL11* | ribosomal protein of the large subunit, mitochondrial |
| *YDL222C* | *YDL222C* | strong similarity to hypothetical protein YNL194c and similarity to YML052w |
| *YDL230W* | *PTP1* | protein tyrosine phosphatase |
| *YDL239C* | *ADY3* | hypothetical protein |
| *YDR011W* | *SNQ2* | multidrug resistance protein |
| *YDR012W* | *RPL4B* | ribosomal protein L4.e.B |
| *YDR014W* | *RAD61* | weak similarity to chicken neurofilament triplet M protein |
| *YDR019C* | *GCV1* | glycine decarboxylase, subunit T |
| *YDR027C* | *LUV1* | interacts with the beta-tubulin binding protein Rbl2p |
| *YDR033W* | *MRH1* | membrane protein related to Hsp30p |
| *YDR036C* | *MRP5* | similarity to enoyl CoA hydratase |
| *YDR041W* | *RSM10* | component of the mitochondrial ribosomal small subunit |
| *YDR049W* | *YDR049W* | similarity to C.elegans K06H7.3 protein |
| *YDR061W* | *YDR061W* | similarity to E.coli modF and photorepair protein phrA |
| *YDR064W* | *RPS13* | ribosomal protein |
| *YDR070C* | *YDR070C* | hypothetical protein |
| *YDR116C* | *MRPL1* | similarity to bacterial ribosomal L1 proteins |
| *YDR146C* | *SWI5* | transcription factor |
| *YDR148C* | *KGD2* | 2-oxoglutarate dehydrogenase complex E2 component |
| *YDR155C* | *CPH1* | cyclophilin (peptidylprolyl isomerase) |
| *YDR174W* | *HMO1* | Non-histone protein |
| *YDR175C* | *RSM24* | component of the mitochondrial ribosomal small subunit |
| *YDR178W* | *SDH4* | succinate dehydrogenase membrane anchor subunit for sdh2p |
| *YDR194C* | *MSS116* | RNA helicase of the DEAD box family, mitochondrial |
| *YDR195W* | *REF2* | RNA 3'-end formation protein |
| *YDR204W* | *COQ4* | responsible for restoring ubiquinone biosynthesis in coq4 mutant |
| *YDR224C* | *HTB1* | histone H2B |
| *YDR225W* | *HTA1* | histone H2A |
| *YDR231C* | *COX20* | in the maturation and assembly of cytochrome oxidase involved protein |
| *YDR232W* | *HEM1* | 5-aminolevulinate synthase |
| *YDR234W* | *LYS4* | homoaconitase |
| *YDR237W* | *MRPL7* | ribosomal protein of the large subunit, mitochondrial |
| *YDR258C* | *HSP78* | heat shock protein of clpb family of ATP-dependent proteases, mitochondrial |
| *YDR285W* | *ZIP1* | synaptonemal complex protein |
| *YDR296W* | *MHR1* | Involved in mitochondrial homologous DNA recombination |
| *YDR298C* | *ATP5* | F1F0-ATPase complex, OSCP subunit |
| *YDR310C* | *SUM1* | suppressor of SIR mutations |
| *YDR322C-A* | *TIM11* | subunit E of the dimeric form of mitochondrial F1F0-ATPase |
| *YDR322W* | *MRPL35* | ribosomal protein YmL35 of the large subunit, mitochondrial |
| *YDR337W* | *MRPS28* | ribosomal protein of the small subunit, mitochondrial |
| *YDR342C* | *HXT7* | high-affinity hexose transporter |
| *YDR343C* | *HXT6* | high-affinity hexose transporter |
| *YDR345C* | *HXT3* | low-affinity hexose transporter |
| *YDR347W* | *MRP1* | ribosomal protein of the small subunit, mitochondrial |
| *YDR376W* | *ARH1* | mitochondrial protein with similarity to human adrenodoxin reductase and ferredoxin-NADP+ reductase |
| *YDR377W* | *ATP17* | ATP synthase complex, subunit f |
| *YDR393W* | *SHE9* | weak similarity to rabbit trichohyalin |
| *YDR406W* | *PDR15* | ATP-binding cassette transporter family member |
| *YDR430C* | *CYM1* | similarity to C.perfringens hypothetical hypA protein |
| *YDR450W* | *RPS18A* | ribosomal protein S18.e.c4 |
| *YDR462W* | *MRPL28* | ribosomal protein of the large subunit (YmL28), mitochondrial |
| *YDR471W* | *RPL27B* | 60S large subunit ribosomal protein |
| *YDR494W* | *RSM28* | weak similarity to hypothetical protein D. melanogaster |
| *YDR495C* | *VPS3* | vacuolar sorting protein |
| *YDR511W* | *ACN9* | weak similarity to C. elegans protein F25H9.7 and to the human complement 3 precursor |
| *YDR513W* | *TTR1* | glutaredoxin |
| *YDR529C* | *QCR7* | ubiquinol--cytochrome-c reductase subunit 7 |
| *YDR536W* | *STL1* | member of the sugar permease family |
| *YEL002C* | *WBP1* | oligosaccharyl transferase beta subunit precursor |
| *YEL020W-A* | *TIM9* | essential subunit of the TIM22-complex for mitochondrial protein import |
| *YEL024W* | *RIP1* | ubiquinol--cytochrome-c reductase iron-sulfur protein precursor |
| *YEL030W* | *ECM10* | heat shock protein of HSP70 family |
| *YEL031W* | *SPF1* | P-type ATPase |
| *YEL039C* | *CYC7* | cytochrome-c isoform 2 |
| *YEL052W* | *AFG1* | ATPase family gene |
| *YEL061C* | *CIN8* | kinesin-related protein |
| *YER004W* | *YER004W* | similarity to hypothetical E.coli and C.elegans proteins |
| *YER008C* | *SEC3* | component of exocyst complex |
| *YER014W* | *HEM14* | protoporphyrinogen oxidase, mitochondrial |
| *YER017C* | *AFG3* | protease of the SEC18/CDC48/PAS1 family of ATPases (AAA) |
| *YER024W* | *YAT2* | carnitine O-acetyltransferase |
| *YER048W-A* | *YER048W-A* | similarity to D. melanogaster protein |
| *YER050C* | *RSM18* | component of the mitochondrial ribosomal small subunit |
| *YER069W* | *ARG5,6* | acetylglutamate kinase and acetylglutamyl-phosphate reductase |
| *YER073W* | *ALD5* | aldehyde dehydrogenase (NAD+), mitochondrial |
| *YER074W* | *RPS24A* | 40s small subunit ribosomal protein S24.e |
| *YER080W* | *YER080W* | hypothetical protein |
| *YER086W* | *ILV1* | anabolic serine and threonine dehydratase precursor |
| *YER087W* | *YER087W* | similarity to E.coli prolyl-tRNA synthetase |
| *YER096W* | *SHC1* | sporulation specific protein |
| *YER102W* | *RPS8B* | ribosomal protein S8.e |
| *YER117W* | *RPL23B* | ribosomal protein L23.e |
| *YER120W* | *SCS2* | required for inositol metabolism |
| *YER141W* | *COX15* | cytochrome oxidase assembly factor |
| *YER154W* | *OXA1* | cytochrome oxidase biogenesis protein |
| *YER178W* | *PDA1* | pyruvate dehydrogenase (lipoamide) alpha chain precursor |
| *YER182W* | *YER182W* | similarity to hypothetical protein SPAC3A12.08 - S. pombe |
| *YFL005W* | *SEC4* | GTP-binding protein of the ras superfamily |
| *YFL016C* | *MDJ1* | heat shock protein - chaperone |
| *YFL018C* | *LPD1* | dihydrolipoamide dehydrogenase precursor |
| *YFL030W* | *YFL030W* | similarity to several transaminases |
| *YFR011C* | *YFR011C* | ochre suppressor tyr-tRNA |
| *YFR033C* | *QCR6* | ubiquinol--cytochrome-c reductase 17K protein |
| *YFR044C* | *YFR044C* | similarity to hypothetical protein YBR281c |
| *YFR049W* | *YMR31* | ribosomal protein, mitochondrial |
| *YGL008C* | *PMA1* | H+-transporting P-type ATPase, major isoform, plasma membrane |
| *YGL031C* | *RPL24A* | 60S large subunit ribosomal protein L24.e.A |
| *YGL055W* | *OLE1* | stearoyl-CoA desaturase |
| *YGL068W* | *YGL068W* | strong similarity to Cricetus mitochrondial ribosomal L12 protein |
| *YGL076C* | *RPL7A* | 60S large subunit ribosomal protein L7.e.A |
| *YGL092W* | *NUP145* | nuclear pore protein |
| *YGL103W* | *RPL28* | 60S large subunit ribosomal protein L27a.e |
| *YGL107C* | *RMD9* | strong similarity to hypothetical protein YBR238c |
| *YGL119W* | *ABC1* | ubiquinol--cytochrome-c reductase complex assembly protein |
| *YGL129C* | *RSM23* | similarity to S.pombe hypothetical protein SPBC29A3.15C - putative mitochondrial function |
| *YGL147C* | *RPL9A* | ribosomal protein L9.e |
| *YGL156W* | *AMS1* | alpha-mannosidase |
| *YGL187C* | *COX4* | cytochrome-c oxidase chain IV |
| *YGL191W* | *COX13* | cytochrome-c oxidase chain VIa |
| *YGL256W* | *ADH4* | alcohol dehydrogenase IV |
| *YGR012W* | *YGR012W* | similarity to E.nidulans cysteine synthase |
| *YGR027C* | *RPS25A* | ribosomal protein S25.e.c7 |
| *YGR032W* | *GSC2* | 1,3-beta-D-glucan synthase subunit |
| *YGR082W* | *TOM20* | mitochondrial outer membrane import receptor subunit, 20 kD |
| *YGR084C* | *MRP13* | ribosomal protein of the small subunit, mitochondrial |
| *YGR086C* | *PIL1* | strong similarity to hypothetical protein YPL004c |
| *YGR091W* | *PRP31* | pre-mRNA splicing protein |
| *YGR094W* | *VAS1* | valyl-tRNA synthetase |
| *YGR112W* | *SHY1* | SURF homologue protein |
| *YGR130C* | *YGR130C* | weak similarity to myosin heavy chain proteins |
| *YGR132C* | *PHB1* | prohibitin, antiproliferative protein |
| *YGR138C* | *TPO2* | polyamine transport protein |
| *YGR148C* | *RPL24B* | 60S large subunit ribosomal protein L24.e.B |
| *YGR157W* | *CHO2* | phosphatidylethanolamine N-methyltransferase |
| *YGR165W* | *MRPS35* | similarity to PIR:T39444 hypothetical protein SPBC14C8.16c S. pombe |
| *YGR174C* | *CBP4* | ubiquinol--cytochrome-c reductase assembly factor |
| *YGR192C* | *TDH3* | glyceraldehyde-3-phosphate dehydrogenase 3 |
| *YGR193C* | *PDX1* | pyruvate dehydrogenase complex protein X |
| *YGR204W* | *ADE3* | C1-tetrahydrofolate synthase (trifunctional enzyme),cytoplasmic |
| *YGR207C* | *ETF-BETA* | electron-transferring flavoprotein, beta chain |
| *YGR214W* | *RPS0A* | 40S ribosomal protein p40 homolog A |
| *YGR215W* | *RSM27* | strong similarity to hypothetical S. pombe protein |
| *YGR220C* | *MRPL9* | ribosomal protein YmL9, mitochondrial |
| *YGR231C* | *PHB2* | Prohibitin |
| *YGR234W* | *YHB1* | flavohemoglobin |
| *YGR235C* | *YGR235C* | hypothetical protein |
| *YGR238C* | *KEL2* | involved in cell fusion and morphogenesis |
| *YGR240C* | *PFK1* | 6-phosphofructokinase, alpha subunit |
| *YGR244C* | *LSC2* | succinate-CoA ligase beta subunit |
| *YGR254W* | *ENO1* | enolase I (2-phosphoglycerate dehydratase) |
| *YGR281W* | *YOR1* | ATP-binding cassette transporter protein |
| *YGR286C* | *BIO2* | biotin synthetase |
| *YHL004W* | *MRP4* | ribosomal protein of the small subunit, mitochondrial |
| *YHL021C* | *YHL021C* | weak similarity to Pseudomonas gamma-butyrobetaine hydroxylase |
| *YHL030W* | *ECM29* | involved in cell wall biogenesis and architecture |
| *YHR001W-A* | *QCR10* | ubiquinol--cytochrome-c reductase 8.5 kDa subunit |
| *YHR005C-A* | *MRS11* | subunit of the Tim22-complex |
| *YHR007C* | *ERG11* | cytochrome P450 lanosterol 14a-demethylase |
| *YHR008C* | *SOD2* | superoxide dismutase (Mn) precursor, mitochondrial |
| *YHR024C* | *MAS2* | processing peptidase, catalytic 53kDa (alpha) subunit, mitochondrial |
| *YHR031C* | *RRM3* | DNA helicase involved in rDNA replication and Ty1 transposition |
| *YHR037W* | *PUT2* | 1-pyrroline-5-carboxylate dehydrogenase |
| *YHR038W* | *FIL1* | Killed in Mutagen, sensitive to Diepoxybutane and/or Mitomycin C |
| *YHR039C* | *MSC7* | similarity to aldehyde dehydrogenases |
| *YHR051W* | *COX6* | cytochrome-c oxidase subunit VI |
| *YHR063C* | *PAN5* | weak similarity to translational activator CBS2 |
| *YHR117W* | *TOM71* | protein with similarity to Tom70p/Mas70p |
| *YHR119W* | *SET1* | involved in chromatin-mediated gene regulation |
| *YHR141C* | *RPL42B* | ribosomal protein L36a.e |
| *YHR147C* | *MRPL6* | ribosomal protein of the large subunit, mitochondrial |
| *YHR190W* | *ERG9* | farnesyl-diphosphate farnesyltransferase |
| *YHR198C* | *YHR198C* | strong similarity to hypothetical protein YHR199c |
| *YHR199C* | *YHR199C* | strong similarity to hypothetical protein YHR198c |
| *YHR203C* | *RPS4B* | ribosomal protein S4.e.c8 |
| *YHR208W* | *BAT1* | branched chain amino acid aminotransferase, mitochondrial |
| *YIL022W* | *TIM44* | mitochondrial inner membrane import receptor subunit |
| *YIL042C* | *YIL042C* | similarity to rat branched-chain alpha-ketoacid dehydrogenase kinase |
| *YIL051C* | *MMD1* | required for maintenance of mitochondrial DNA |
| *YIL065C* | *FIS1* | protein involved in mitochondrial division |
| *YIL069C* | *RPS24B* | 40S small subunit ribosomal protein S24.e |
| *YIL070C* | *MAM33* | mitochondrial acidic matrix protein |
| *YIL093C* | *RSM25* | weak similarity to S.pombe hypothetical protein SPBC16A3 |
| *YIL094C* | *LYS12* | homo-isocitrate dehydrogenase |
| *YIL098C* | *FMC1* | Formation of Mitochondrial Cytochromes |
| *YIL111W* | *COX5B* | cytochrome-c oxidase chain Vb |
| *YIL124W* | *AYR1* | 1-Acyldihydroxyacetone-phosphate reductase |
| *YIL125W* | *KGD1* | 2-oxoglutarate dehydrogenase complex E1 component |
| *YIL136W* | *OM45* | protein of the outer mitochondrial membrane |
| *YIL149C* | *MLP2* | involved in translocation of macromolecules between the nucleoplasm and the NPC |
| *YIL155C* | *GUT2* | glycerol-3-phosphate dehydrogenase, mitochondrial |
| *YIL157C* | *YIL157C* | weak similarity to PIR:T39578 hypothetical protein SPBC16E9.03c S. pombe |
| *YIR006C* | *PAN1* | actin-cytoskeleton assembly protein |
| *YJL021C* | *BBC1* | shows synthetic fitness defect with bni1 mutants and associates with the Bee1p-Vrp1p-Myo3/5p complex |
| *YJL034W* | *KAR2* | nuclear fusion protein |
| *YJL045W* | *YJL045W* | strong similarity to succinate dehydrogenase flavoprotein |
| *YJL052W* | *TDH1* | glyceraldehyde-3-phosphate dehydrogenase 1 |
| *YJL054W* | *TIM54* | translocase for the insertion of proteins into the mitochondrial inner membrane |
| *YJL060W* | *BNA3* | similarity to kynurenine aminotransferase and glutamine-phenylpyruvate transaminase |
| *YJL063C* | *MRPL8* | ribosomal protein L17, mitochondrial |
| *YJL066C* | *MPM1* | hypothetical protein |
| *YJL096W* | *MRPL49* | ribosomal protein YmL49, mitochondrial |
| *YJL130C* | *URA2* | multifunctional pyrimidine biosynthesis protein |
| *YJL166W* | *QCR8* | ubiquinol--cytochrome-c reductase chain VIII |
| *YJL168C* | *SET2* | enhancer of zeste-like |
| *YJL171C* | *YJL171C* | similarity to YBR162c |
| *YJL177W* | *RPL17B* | 60s large subunit ribosomal protein L17.e |
| *YJL191W* | *RPS14B* | 40S small subunit ribosomal protein S14.e.B |
| *YJL200C* | *YJL200C* | strong similarity to aconitate hydratase |
| *YJL208C* | *NUC1* | nuclease, mitochondrial |
| *YJR009C* | *TDH2* | glyceraldehyde-3-phosphate dehydrogenase 2 |
| *YJR016C* | *ILV3* | dihydroxy-acid dehydratase |
| *YJR042W* | *NUP85* | nuclear pore protein |
| *YJR045C* | *SSC1* | mitochondrial heat shock protein 70-related protein |
| *YJR048W* | *CYC1* | cytochrome-c isoform 1 |
| *YJR059W* | *PTK2* | involved in polyamine uptake |
| *YJR077C* | *MIR1* | phosphate transport protein, mitochondrial (MCF) |
| *YJR080C* | *YJR080C* | hypothetical protein |
| *YJR090C* | *GRR1* | required for glucose repression and for glucose and cation transport |
| *YJR095W* | *SFC1* | succinate-fumarate transporter |
| *YJR101W* | *RSM26* | weak similarity to superoxide dismutases |
| *YJR104C* | *SOD1* | copper-zinc superoxide dismutase |
| *YJR113C* | *RSM7* | similarity to bacterial, chloroplast and mitochondrial ribosomal protein S7 |
| *YJR121W* | *ATP2* | F1F0-ATPase complex, F1 beta subunit |
| *YJR143C* | *PMT4* | dolichyl-phosphate-mannose--protein O-mannosyl transferase |
| *YJR144W* | *MGM101* | mitochondrial genome maintenance protein |
| *YJR145C* | *RPS4A* | ribosomal protein S4.e.c10 |
| *YJR148W* | *BAT2* | branched chain amino acid aminotransferase, cytosolic |
| *YKL016C* | *ATP7* | F1F0-ATPase complex, FO D subunit |
| *YKL027W* | *YKL027W* | similarity to E.coli molybdopterin-converting factor chlN |
| *YKL029C* | *MAE1* | malic enzyme |
| *YKL040C* | *NFU1* | iron homeostasis |
| *YKL085W* | *MDH1* | malate dehydrogenase precursor, mitochondrial |
| *YKL120W* | *OAC1* | similarity to mitochondrial uncoupling proteins (MCF) |
| *YKL135C* | *APL2* | AP-1 complex subunit, beta1-adaptin, 82 KD |
| *YKL141W* | *SDH3* | cytochrome b560 subunit of respiratory complex II |
| *YKL148C* | *SDH1* | succinate dehydrogenase flavoprotein precursor |
| *YKL150W* | *MCR1* | cytochrome-b5 reductase |
| *YKL152C* | *GPM1* | phosphoglycerate mutase |
| *YKL157W* | *APE2* | aminopeptidase yscII |
| *YKL171W* | *YKL171W* | weak similarity to ser/thr protein kinase |
| *YKL192C* | *ACP1* | mitochonrial acyl-carrier protein |
| *YKL195W* | *YKL195W* | similarity to rabbit histidine-rich calcium-binding protein |
| *YKL203C* | *TOR2* | phosphatidylinositol 3-kinase |
| *YKL212W* | *SAC1* | recessive suppressor of secretory defect |
| *YKL215C* | *YKL215C* | similarity to P. aeruginosa hyuA and hyuB |
| *YKL217W* | *JEN1* | carboxylic acid transporter protein |
| *YKR016W* | *YKR016W* | weak similarity to mysoin heavy chain proteins |
| *YKR028W* | *SAP190* | Sit4p-associated protein |
| *YKR049C* | *YKR049C* | hypothetical protein |
| *YKR065C* | *YKR065C* | similarity to hypothetical protein S. pombe |
| *YKR066C* | *CCP1* | cytochrome-c peroxidase precursor |
| *YKR070W* | *YKR070W* | strong similarity to S. pombe phosphatidyl synthase |
| *YKR085C* | *MRPL20* | ribosomal protein YmL20, mitochondrial |
| *YKR095W* | *MLP1* | myosin-like protein related to Uso1p |
| *YLL021W* | *SPA2* | involved in cell polarity |
| *YLL041C* | *SDH2* | succinate dehydrogenase iron-sulfur protein subunit |
| *YLR008C* | *YLR008C* | similarity to hypothetical protein YNL328c |
| *YLR038C* | *COX12* | cytochrome-c oxidase, subunit VIB |
| *YLR056W* | *ERG3* | C-5 sterol desaturase |
| *YLR069C* | *MEF1* | translation elongation factor G, mitochondrial |
| *YLR075W* | *RPL10* | 60S large subunit ribosomal protein |
| *YLR089C* | *YLR089C* | strong similarity to alanine transaminases |
| *YLR106C* | *MDN1* | similarity to Kaposi's sarcoma-associated herpes-like virus ORF73 homolog gene |
| *YLR108C* | *YLR108C* | strong similarity to YDR132c |
| *YLR131C* | *ACE2* | metallothionein expression activator |
| *YLR163C* | *MAS1* | mitochondrial processing peptidase |
| *YLR174W* | *IDP2* | isocitrate dehydrogenase, cytosolic |
| *YLR188W* | *MDL1* | ATP-binding cassette transporter family member |
| *YLR201C* | *YLR201C* | similarity to hypothetical S. pombe protein |
| *YLR239C* | *LIP2* | lipoic acid ligase |
| *YLR259C* | *HSP60* | heat shock protein - chaperone, mitochondrial |
| *YLR295C* | *ATP14* | F1F0-ATPase complex, subunit h |
| *YLR304C* | *ACO1* | aconitate hydratase |
| *YLR309C* | *IMH1* | involved in vesicular transport |
| *YLR312W-A* | *MRPL15* | ribosomal protein of the large subunit, mitochondrial |
| *YLR325C* | *RPL38* | 60S large subunit ribosomal protein |
| *YLR342W* | *FKS1* | 1,3-beta-D-glucan synthase, catalytic subunit |
| *YLR348C* | *DIC1* | dicarboxylate carrier protein |
| *YLR355C* | *ILV5* | ketol-acid reducto-isomerase |
| *YLR369W* | *SSQ1* | mitochondrial heat shock protein 70 |
| *YLR393W* | *ATP10* | F1F0 ATPase complex assembly protein |
| *YLR395C* | *COX8* | cytochrome-c oxidase chain VIII |
| *YLR433C* | *CNA1* | calcineurin B, catalytic subunit |
| *YLR439W* | *MRPL4* | ribosomal protein, mitochondrial |
| *YLR441C* | *RPS1A* | ribosomal protein S3a.e |
| *YML002W* | *YML002W* | hypothetical protein |
| *YML008C* | *ERG6* | S-adenosyl-methionine delta-24-sterol-c-methyltransferase |
| *YML026C* | *RPS18B* | ribosomal protein S18.e.c13 |
| *YML030W* | *YML030W* | weak similarity to CG9921 D. melanogaster |
| *YML042W* | *CAT2* | carnitine O-acetyltransferase |
| *YML054C* | *CYB2* | lactate dehydrogenase cytochrome b2 |
| *YML063W* | *RPS1B* | ribosomal protein S3a.e |
| *YML072C* | *YML072C* | similarity to YOR3141c and YNL087w |
| *YML078W* | *CPR3* | cyclophilin (peptidylprolyl isomerase), mitochondrial |
| *YML081C-A* | *ATP18* | gene for subunit i/j of the mitochondrial F1F0-ATP synthase |
| *YML086C* | *ALO1* | D-arabinono-1,4-lactone oxidase |
| *YML091C* | *RPM2* | ribonuclease P precursor, mitochondrial |
| *YML103C* | *NUP188* | nuclear pore protein |
| *YML107C* | *YML107C* | hypothetical protein |
| *YML110C* | *COQ5* | ubiquinone biosynthesis, methyltransferase |
| *YML120C* | *NDI1* | NADH-ubiquinone-6 oxidoreductase |
| *YML123C* | *PHO84* | high-affinity inorganic phosphate/H+ symporter |
| *YMR008C* | *PLB1* | phospholipase B (lysophospholipase) |
| *YMR011W* | *HXT2* | high-affinity hexose transporter |
| *YMR024W* | *MRPL3* | ribosomal protein of the large subunit, mitochondrial |
| *YMR056C* | *AAC1* | ADP/ATP carrier protein (MCF) |
| *YMR062C* | *ECM40* | acetylornithine acetyltransferase |
| *YMR072W* | *ABF2* | high mobility group protein |
| *YMR083W* | *ADH3* | alcohol dehydrogenase III |
| *YMR089C* | *YTA12* | protease of the SEC18/CDC48/PAS1 family of ATPases (AAA) |
| *YMR108W* | *ILV2* | acetolactate synthase |
| *YMR110C* | *YMR110C* | similarity to aldehyde dehydrogenase |
| *YMR115W* | *YMR115W* | similarity to YKL133c |
| *YMR124W* | *YMR124W* | weak similarity to YLR031w |
| *YMR145C* | *NDE1* | mitochondrial cytosolically directed NADH dehydrogenase |
| *YMR158W* | *MRPS8* | weak similarity to E.coli ribosomal S8 protein |
| *YMR162C* | *DNF3* | similarity to ATPases |
| *YMR165C* | *SMP2* | involved in plasmid maintenance, respiration and cell proliferation |
| *YMR186W* | *HSC82* | heat shock protein |
| *YMR189W* | *GCV2* | glycine decarboxylase P subunit |
| *YMR193W* | *MRPL24* | ribosomal protein of the large subunit, mitochondrial |
| *YMR203W* | *TOM40* | forms the hydrophilic channel of the mitochondrial import pore for preproteins |
| *YMR241W* | *YHM2* | yeast suppressor gene of HM (mitochondrial histone) mutant (abf2) |
| *YMR256C* | *COX7* | cytochrome-c oxidase, subunit VII |
| *YMR266W* | *RSN1* | similarity to A.thaliana hyp1 protein |
| *YMR286W* | *MRPL33* | ribosomal protein of the large subunit, mitochondrial |
| *YMR296C* | *LCB1* | serine C-palmitoyltransferase subunit |
| *YMR301C* | *ATM1* | ATP-binding cassette transporter protein, mitochondrial |
| *YMR302C* | *PRP12* | involved in early maturation of pre-rRNA |
| *YMR307W* | *GAS1* | glycophospholipid-anchored surface glycoprotein |
| *YNL005C* | *MRP7* | ribosomal protein YmL2 precursor, mitochondrial |
| *YNL009W* | *IDP3* | isocitrate dehydrogenase, NADP-dependent |
| *YNL026W* | *YNL026W* | similarity to S.pombe hypothetical protein |
| *YNL030W* | *HHF2* | histone H4 |
| *YNL031C* | *HHT2* | histone H3 |
| *YNL037C* | *IDH1* | isocitrate dehydrogenase (NAD+) subunit 1, mitochondrial |
| *YNL040W* | *YNL040W* | weak similarity to M.genitalium alanine--tRNA ligase |
| *YNL052W* | *COX5A* | cytochrome-c oxidase chain V.A precursor |
| *YNL055C* | *POR1* | mitochondrial outer membrane porin |
| *YNL058C* | *YNL058C* | similarity to YIL117c |
| *YNL067W* | *RPL9B* | ribosomal protein L9.e.c14 |
| *YNL070W* | *TOM7* | mitochondrial outer membrane import receptor subunit, 7 kD |
| *YNL071W* | *LAT1* | dihydrolipoamide S-acetyltransferase |
| *YNL100W* | *YNL100W* | hypothetical protein |
| *YNL104C* | *LEU4* | 2-isopropylmalalate synthase |
| *YNL121C* | *TOM70* | mitochondrial outer membrane specialized import receptor |
| *YNL131W* | *TOM22* | mitochondrial outer membrane import receptor complex subunit |
| *YNL137C* | *NAM9* | ribosomal protein, mitochondrial |
| *YNL142W* | *MEP2* | high affinity low capacity ammonia permease |
| *YNL163C* | *RIA1* | translation elongation factor eEF4 |
| *YNL168C* | *YNL168C* | similarity to C.elegans ZK688.3 protein and E.coli hpcEp |
| *YNL169C* | *PSD1* | phosphatidylserine decarboxylase 1 |
| *YNL178W* | *RPS3* | ribosomal protein S3.e |
| *YNL185C* | *MRPL19* | ribosomal protein of the large subunit, mitochondrial |
| *YNL197C* | *WHI3* | involved in regulation of cell size |
| *YNL239W* | *LAP3* | member of the GAL regulon |
| *YNL248C* | *RPA49* | DNA-directed RNA polymerase A (I) chain, 46 kDa |
| *YNL252C* | *MRPL17* | ribosomal protein of the large subunit (YmL30), mitochondrial |
| *YNL258C* | *DSL1* | hypothetical protein |
| *YNL262W* | *POL2* | DNA-directed DNA polymerase epsilon, catalytic subunit A |
| *YNL284C* | *MRPL10* | ribosomal protein of the large subunit, mitochondrial |
| *YNL306W* | *MRPS18* | ribosomal protein of the small subunit, mitochondrial |
| *YNL309W* | *STB1* | Sin3p binding protein |
| *YNL315C* | *ATP11* | F1F0-ATPase complex assembly protein |
| *YNL323W* | *LEM3* | similarity to Ycx1p |
| *YNR001C* | *CIT1* | citrate (si)-synthase, mitochondrial |
| *YNR017W* | *MAS6* | mitochondrial inner membrane import translocase subunit |
| *YNR018W* | *YNR018W* | similarity to TRCDSEMBL:SPAC1565_1 hypothetical protein S. pombe |
| *YNR022C* | *MRPL50* | weak similarity to protein phosphatases |
| *YOL021C* | *DIS3* | 3'->5' exoribonuclease required for 3' end formation of 5.8S rRNA |
| *YOL027C* | *MDM38* | similarity to YPR125w |
| *YOL040C* | *RPS15* | 40S small subunit ribosomal protein |
| *YOL053W* | *YOL053W* | hypothetical protein |
| *YOL070C* | *YOL070C* | hypothetical protein |
| *YOL071W* | *EMI5* | similarity to hypothetical S. pombe protein |
| *YOL077W-A* | *ATP19* | subunit K of the dimeric form of mitochondrial F1F0-ATP synthase |
| *YOL109W* | *ZEO1* | weak similarity to G.hirsutum embryonic abundant protein D-7 |
| *YOL115W* | *TRF4* | topoisomerase I-related protein |
| *YOL140W* | *ARG8* | acetylornithine aminotransferase |
| *YOR020C* | *HSP10* | chaperonin, mitochondrial |
| *YOR027W* | *STI1* | stress-induced protein |
| *YOR035C* | *SHE4* | required for mother cell-specific gene expression |
| *YOR040W* | *GLO4* | glyoxalase II (hydroxyacylglutathione hydrolase) |
| *YOR063W* | *RPL3* | 60S large subunit ribosomal protein L3.e |
| *YOR065W* | *CYT1* | cytochrome-c1 |
| *YOR092W* | *ECM3* | involved in cell wall biogenesis and architecture |
| *YOR096W* | *RPS7A* | ribosomal protein |
| *YOR100C* | *CRC1* | mitochondrial carnitine carrier, member of the mitochondrial carrier (MCF) family |
| *YOR108W* | *LEU9* | strong similarity to Leu4p |
| *YOR131C* | *YOR131C* | weak similarity to E.coli hypothetical 27K protein |
| *YOR136W* | *IDH2* | isocitrate dehydrogenase (NAD+) subunit 2, mitochondrial |
| *YOR142W* | *LSC1* | succinate-CoA ligase alpha subunit |
| *YOR153W* | *PDR5* | pleiotropic drug resistance protein |
| *YOR158W* | *PET123* | ribosomal protein, mitochondrial |
| *YOR176W* | *HEM15* | ferrochelatase precursor |
| *YOR187W* | *TUF1* | translation elongation factor TU, mitochondrial |
| *YOR201C* | *PET56* | rRNA (guanosine-2'-O-)-methyltransferase |
| *YOR211C* | *MGM1* | dynamin-like protein |
| *YOR215C* | *YOR215C* | similarity to M.xanthus hypothetical protein |
| *YOR221C* | *MCT1* | malonyl-CoA:ACP transferase |
| *YOR222W* | *ODC2* | similarity to ADP/ATP carrier proteins |
| *YOR230W* | *WTM1* | transcriptional modulator |
| *YOR232W* | *MGE1* | heat shock protein - chaperone |
| *YOR249C* | *APC5* | component of the anaphase-promoting complex |
| *YOR251C* | *YOR251C* | similarity to thiosulfate sulfurtransferases |
| *YOR254C* | *SEC63* | ER protein-translocation complex subunit |
| *YOR271C* | *YOR271C* | strong similarity to Rattus tricarboxylate carrier |
| *YOR286W* | *YOR286W* | similarity to D.melanogaster heat shock protein 67B2 |
| *YOR317W* | *FAA1* | long-chain-fatty-acid--CoA ligase |
| *YOR334W* | *MRS2* | RNA splicing protein and member of the mitochondrial carrier family (MCF) |
| *YOR354C* | *MSC6* | weak similarity to genomic sequence for A. thaliana |
| *YOR356W* | *YOR356W* | strong similarity to human electron transfer flavoprotein-ubiquinone oxidoreductase |
| *YOR374W* | *ALD4* | aldehyde dehydrogenase, mitochondrial |
| *YPL004C* | *LSP1* | strong similarity to YGR086c |
| *YPL022W* | *RAD1* | component of the nucleotide excision repairosome |
| *YPL036W* | *PMA2* | H+-transporting P-type ATPase, minor isoform, plasma membrane |
| *YPL043W* | *NOP4* | nucleolar protein |
| *YPL058C* | *PDR12* | multidrug resistance transporter |
| *YPL059W* | *GRX5* | member of the subfamily of yeast glutaredoxins (Grx3, Grx4, and Grx5) |
| *YPL063W* | *TIM50* | similarity to hypothetical protein YLR019w, YLL010c and S.pombe hypothetical protein SPAC2F7.02c |
| *YPL078C* | *ATP4* | F1F0-ATPase complex, F0 subunit B |
| *YPL090C* | *RPS6A* | ribosomal protein S6.e |
| *YPL091W* | *GLR1* | glutathione reductase (NADPH) |
| *YPL097W* | *MSY1* | tyrosyl-tRNA synthetase |
| *YPL099C* | *YPL099C* | weak similarity to Sulfolobus hypothetical protein |
| *YPL104W* | *MSD1* | aspartate--tRNA ligase, mitochondrial |
| *YPL106C* | *SSE1* | heat shock protein of HSP70 family |
| *YPL118W* | *MRP51* | mitochondrial ribosomal protein of the small subunit |
| *YPL120W* | *VPS30* | involved in vacuolare protein sorting and autophagy |
| *YPL131W* | *RPL5* | 60S large subunit ribosomal protein L5.e |
| *YPL132W* | *COX11* | cytochrome-c oxidase assembly protein |
| *YPL134C* | *ODC1* | similarity to ADP,ATP carrier proteins |
| *YPL167C* | *REV3* | DNA-directed DNA polymerase zeta subunit |
| *YPL173W* | *MRPL40* | ribosomal protein of the large subunit (YmL40), mitochondrial |
| *YPL174C* | *NIP100* | component of the dynactin complex |
| *YPL188W* | *POS5* | similarity to Utr1p and YEL041w |
| *YPL198W* | *RPL7B* | 60S large subunit ribosomal protein |
| *YPL215W* | *CBP3* | required for assembly of cytochrome bc1 complex |
| *YPL262W* | *FUM1* | fumarate hydratase |
| *YPL265W* | *DIP5* | dicarboxylic amino acid permease |
| *YPL270W* | *MDL2* | ATP-binding cassette (ABC) transporter family member |
| *YPL271W* | *ATP15* | F1F0-ATPase complex, F1 epsilon subunit |
| *YPR001W* | *CIT3* | citrate (si)-synthase, mitochondrial |
| *YPR002W* | *PDH1* | similarity to B.subtilis mmgE protein |
| *YPR004C* | *YPR004C* | strong similarity to electron transfer flavoproteins alpha chain |
| *YPR006C* | *ICL2* | non-functional isocitrate lyase |
| *YPR020W* | *ATP20* | subunit G of the dimeric form of mitochondrial F1F0-ATP Synthase |
| *YPR024W* | *YME1* | protease of the SEC18/CDC48/PAS1 family of ATPases (AAA) |
| *YPR026W* | *ATH1* | acid trehalase, vacuolar |
| *YPR035W* | *GLN1* | glutamate--ammonia ligase |
| *YPR052C* | *NHP6A* | nonhistone chromosomal protein related to mammalian HMG1 |
| *YPR058W* | *YMC1* | mitochondrial carrier protein (MCF) |
| *YPR067W* | *ISA2* | mitochondrial protein required for iron metabolism |
| *YPR080W* | *TEF1* | translation elongation factor eEF1 alpha-A chain, cytosolic |
| *YPR100W* | *MRPL51* | weak similarity to C.elegans hypothetical protein CEC25A1 |
| *YPR115W* | *YPR115W* | similarity to probable transcription factor Ask10p, and to YNL047c and YIL105c |
| *YPR125W* | *MRS7* | suppressor of mrs2-1 mutation |
| *YPR133W-A* | *TOM5* | mitochondrial outer membrane protein |
| *YPR135W* | *CTF4* | DNA-directed DNA polymerase alpha-binding protein |
| *YPR164W* | *KIM3* | drug resistance |
| *YPR183W* | *DPM1* | dolichyl-phosphate beta-D-mannosyltransferase |
| *YPR191W* | *QCR2* | ubiquinol--cytochrome-c reductase 40KD chain II |
