# Supplementary material for: Integrative Analysis of the Mitochondrial Proteome in Yeast
Source: PLoS Biol. 2004 Jun 15;2(6):e160. doi: 10.1371/journal.pbio.0020160 (PMC423137; doi:10.1371/journal.pbio.0020160)
Supplement: Table S3 — (828 KB DOC). [file pbio.0020160.st003.doc]

**Table S2.** Human orthologs of yeast mitochondria-related proteins.

| Yeast  ORF | Gene | Known mito  yeast^a^ | MitoP2  score | Human  ID | Known mito  human^b^ | BLAST  e-value^c^ | Alignment  length (%)^d^ |
| --- | --- | --- | --- | --- | --- | --- | --- |
| *Q0045* | *COX1* | yes | 97 | P00395 | yes | 0 | 98 |
| *Q0085* | *ATP6* | yes | 97 | P00846 | yes | 5.00E-047 | 97 |
| *Q0105* | *COB* | yes | 97 | P00156 | yes | 1.00E-137 | 97 |
| *Q0130* | *OLI1* | yes | 97 | P05496 | yes | 9.00E-017 | 53 |
| *Q0130* | *OLI1* | yes | 97 | P48201 | yes | 9.00E-017 | 53 |
| *Q0130* | *OLI1* | yes | 97 | Q06055 | yes | 9.00E-017 | 53 |
| *Q0250* | *COX2* | yes | 97 | P00403 | yes | 2.00E-085 | 95 |
| *Q0275* | *COX3* | yes | 97 | P00414 | yes | 1.00E-089 | 99 |
| *YAL012W* | *CYS3* | - | 97 | P32929 | - | 6.00E-079 | 98 |
| *YAL035W* | *FUN12* | - | 97 | O60841 | - | 1.00E-116 | 59 |
| *YAL039C* | *CYC3* | yes | 98 | P53701 | yes | 5.00E-088 | 86 |
| *YAL044C* | *GCV3* | yes | 98 | P23434 | yes | 1.00E-058 | 73 |
| *YAL054C* | *ACS1* | - | 95 | Q9NR19 | - | 1.00E-172 | 95 |
| *YAL062W* | *GDH3* | - | 93 | P00367 | yes | 1.00E-150 | 88 |
| *YBL004W* | *UTP20* | - | 96 | O75691 | - | 1.00E-127 | 14 |
| *YBL013W* | *FMT1* | yes | 98 | Q96DP5 | yes | 4.00E-074 | 78 |
| *YBL022C* | *PIM1* | yes | 98 | P36776 | yes | 1.00E-147 | 88 |
| *YBL027W* | *RPL19B* | - | 95 | P14118 | - | 1.00E-069 | 98 |
| *YBL038W* | *MRPL16* | yes | 98 | Q9BYD0 | yes | 2.00E-070 | 80 |
| *YBL080C* | *PET112* | yes | 98 | O75879 | yes | 0 | 96 |
| *YBL087C* | *RPL23A* | - | 96 | P23131 | yes | 5.00E-054 | 98 |
| *YBL098W* | *BNA4* | - | 96 | O15229 | - | 1.00E-135 | 88 |
| *YBL099W* | *ATP1* | yes | 98 | P25705 | yes | 0 | 93 |
| *YBR003W* | *COQ1* | yes | 98 | Q9Y2W5 | - | 3.00E-082 | 75 |
| *YBR024W* | *SCO2* | yes | 98 | O75880 | yes | 4.00E-095 | 83 |
| *YBR029C* | *CDS1* | yes | 97 | O95674 | yes | 1.00E-174 | 97 |
| *YBR031W* | *RPL4A* | - | 94 | P36578 | - | 1.00E-153 | 92 |
| *YBR039W* | *ATP3* | yes | 98 | P36542 | yes | 1.00E-104 | 93 |
| *YBR084C-A* | *RPL19A* | - | 95 | P14118 | - | 1.00E-069 | 98 |
| *YBR084W* | *MIS1* | yes | 98 | P11586 | yes | 0 | 100 |
| *YBR084W* | *MIS1* | yes | 98 | Q8WVW0 | - | 0 | 100 |
| *YBR084W* | *MIS1* | yes | 98 | Q9UFU8 | - | 0 | 99 |
| *YBR121C* | *GRS1* | yes | 95 | P41250 | - | 0 | 95 |
| *YBR127C* | *VMA2* | - | 96 | P15313 | - | 0 | 92 |
| *YBR127C* | *VMA2* | - | 96 | P21281 | - | 0 | 91 |
| *YBR136W* | *MEC1* | - | 97 | Q13315 | - | 0 | 77 |
| *YBR136W* | *MEC1* | - | 97 | Q13535 | - | 0 | 71 |
| *YBR136W* | *MEC1* | - | 97 | Q9Y4A5 | - | 0 | 72 |
| *YBR146W* | *MRPS9* | yes | 98 | P82933 | yes | 2.00E-064 | 72 |
| *YBR218C* | *PYC2* | - | 95 | P11498 | yes | 0 | 97 |
| *YBR221C* | *PDB1* | yes | 98 | Q9UFK3 | - | 1.00E-127 | 100 |
| *YBR263W* | *SHM1* | yes | 98 | P34896 | yes | 0 | 98 |
| *YBR263W* | *SHM1* | yes | 98 | P34897 | yes | 0 | 94 |
| *YBR291C* | *CTP1* | yes | 91 | P12236 | yes | 3.00E-075 | 100 |
| *YCL017C* | *NFS1* | yes | 98 | Q9Y697 | yes | 1.00E-093 | 92 |
| *YCL033C* | *YCL033C* | - | 96 | Q9Y3D2 | - | 4.00E-047 | 74 |
| *YCL057W* | *PRD1* | yes | 97 | Q9BYT8 | yes | 0 | 94 |
| *YCL057W* | *PRD1* | yes | 97 | P52888 | - | 0 | 97 |
| *YCL057W* | *PRD1* | yes | 97 | Q9BQD0 | - | 0 | 80 |
| *YCR005C* | *CIT2* | - | 95 | O75390 | yes | 0 | 95 |
| *YCR012W* | *PGK1* | - | 93 | P00558 | - | 0 | 99 |
| *YCR012W* | *PGK1* | - | 93 | P07205 | - | 0 | 100 |
| *YCR012W* | *PGK1* | - | 93 | Q8NI87 | - | 0 | 100 |
| *YCR047C* | *BUD23* | - | 95 | O43709 | - | 9.00E-051 | 100 |
| *YCR053W* | *THR4* | - | 96 | Q9H6P9 | - | 7.00E-062 | 71 |
| *YCR071C* | *IMG2* | yes | 98 | Q13405 | yes | 7.00E-033 | 55 |
| *YDL004W* | *ATP16* | yes | 98 | P30049 | yes | 8.00E-041 | 62 |
| *YDL015C* | *TSC13* | - | 95 | Q9NZ01 | - | 5.00E-081 | 96 |
| *YDL033C* | *YDL033C* | - | 97 | O75648 | - | 1.00E-140 | 90 |
| *YDL066W* | *IDP1* | yes | 98 | P48735 | yes | 0 | 96 |
| *YDL078C* | *MDH3* | - | 93 | P00338 | - | 1.00E-126 | 94 |
| *YDL120W* | *YFH1* | yes | 98 | Q16595 | yes | 2.00E-027 | 40 |
| *YDL140C* | *RPO21* | - | 95 | O14802 | - | 0 | 98 |
| *YDL140C* | *RPO21* | - | 95 | P24928 | - | 0 | 90 |
| *YDL141W* | *BPL1* | - | 95 | P50747 | yes | 1.00E-142 | 54 |
| *YDL171C* | *GLT1* | - | 97 | Q12882 | - | 3.00E-097 | 49 |
| *YDL178W* | *DLD2* | yes | 97 | Q8N465 | - | 1.00E-156 | 89 |
| *YDL185W* | *TFP1* | - | 97 | P38607 | - | 1.00E-153 | 99 |
| *YDL202W* | *MRPL11* | yes | 98 | Q96B80 | - | 9.00E-059 | 66 |
| *YDL217C* | *TIM22* | yes | 93 | Q9Y584 | yes | 1.00E-031 | 74 |
| *YDR012W* | *RPL4B* | - | 94 | P36578 | - | 1.00E-153 | 92 |
| *YDR019C* | *GCV1* | yes | 98 | P48728 | yes | 1.00E-130 | 93 |
| *YDR036C* | *MRP5* | - | 96 | Q92931 | - | 1.00E-106 | 85 |
| *YDR041W* | *RSM10* | yes | 98 | P82664 | yes | 2.00E-035 | 56 |
| *YDR062W* | *LCB2* | - | 95 | O15270 | - | 1.00E-108 | 89 |
| *YDR064W* | *RPS13* | - | 95 | Q02546 | - | 8.00E-071 | 99 |
| *YDR069C* | *DOA4* | - | 95 | P40818 | - | 7.00E-091 | 47 |
| *YDR115W* | *YDR115W* | yes | 98 | Q9BQ48 | yes | 3.00E-016 | 54 |
| *YDR148C* | *KGD2* | yes | 98 | P36957 | yes | 1.00E-141 | 96 |
| *YDR204W* | *COQ4* | yes | 98 | Q9Y3A0 | - | 1.00E-106 | 89 |
| *YDR225W* | *HTA1* | - | 92 | P02261 | - | 3.00E-044 | 81 |
| *YDR225W* | *HTA1* | - | 92 | P20670 | - | 3.00E-044 | 81 |
| *YDR232W* | *HEM1* | yes | 98 | P22557 | yes | 1.00E-088 | 75 |
| *YDR268W* | *MSW1* | yes | 98 | Q9UGM6 | yes | 1.00E-110 | 96 |
| *YDR294C* | *DPL1* | - | 95 | O95470 | - | 1.00E-103 | 93 |
| *YDR294C* | *DPL1* | - | 95 | Q9ULG8 | - | 1.00E-103 | 91 |
| *YDR298C* | *ATP5* | yes | 98 | P48047 | yes | 2.00E-070 | 86 |
| *YDR337W* | *MRPS28* | yes | 98 | P82914 | yes | 4.00E-052 | 72 |
| *YDR341C* | *YDR341C* | - | 91 | P54136 | - | 0 | 97 |
| *YDR341C* | *YDR341C* | - | 91 | Q96FU5 | - | 0 | 90 |
| *YDR405W* | *MRP20* | yes | 98 | Q16540 | yes | 2.00E-050 | 89 |
| *YDR430C* | *CYM1* | - | 95 | O95204 | - | 0 | 98 |
| *YDR430C* | *CYM1* | - | 95 | Q9UPP8 | - | 0 | 99 |
| *YDR450W* | *RPS18A* | - | 96 | P25232 | - | 8.00E-063 | 97 |
| *YDR477W* | *SNF1* | - | 92 | P54646 | - | 1.00E-086 | 90 |
| *YDR511W* | *ACN9* | yes | 98 | Q9NRP4 | - | 2.00E-035 | 90 |
| *YDR519W* | *FPR2* | - | 91 | P26885 | - | 4.00E-039 | 99 |
| *YDR529C* | *QCR7* | yes | 98 | P14927 | yes | 2.00E-030 | 68 |
| *YEL024W* | *RIP1* | yes | 98 | P47985 | yes | 3.00E-086 | 98 |
| *YEL050C* | *RML2* | yes | 98 | Q9Y311 | - | 1.00E-071 | 62 |
| *YER017C* | *AFG3* | yes | 98 | Q9Y4W6 | yes | 1.00E-142 | 80 |
| *YER050C* | *RSM18* | yes | 98 | Q9Y3D5 | yes | 1.00E-016 | 35 |
| *YER061C* | *CEM1* | yes | 97 | Q9NWU1 | - | 1.00E-125 | 94 |
| *YER069W* | *"ARG5,6"* | yes | 97 | Q8N159 | - | 0 | 79 |
| *YER073W* | *ALD5* | yes | 97 | P05091 | yes | 0 | 99 |
| *YER073W* | *ALD5* | yes | 97 | P30837 | yes | 0 | 99 |
| *YER073W* | *ALD5* | yes | 97 | O94788 | - | 0 | 98 |
| *YER073W* | *ALD5* | yes | 97 | P00352 | - | 0 | 99 |
| *YER073W* | *ALD5* | yes | 97 | Q8WX76 | - | 0 | 99 |
| *YER074W* | *RPS24A* | - | 96 | P16632 | - | 2.00E-040 | 98 |
| *YER078C* | *YER078C* | - | 95 | Q9BV27 | - | 1.00E-147 | 90 |
| *YER078C* | *YER078C* | - | 95 | Q9NQH7 | - | 1.00E-147 | 90 |
| *YER117W* | *RPL23B* | - | 96 | P23131 | yes | 5.00E-054 | 98 |
| *YER141W* | *COX15* | yes | 98 | Q9NTN0 | yes | 1.00E-144 | 91 |
| *YER141W* | *COX15* | yes | 98 | O75878 | - | 1.00E-144 | 91 |
| *YER154W* | *OXA1* | yes | 98 | Q15070 | yes | 1.00E-114 | 85 |
| *YER168C* | *CCA1* | yes | 98 | Q96Q11 | - | 1.00E-119 | 86 |
| *YER178W* | *PDA1* | yes | 98 | P29803 | yes | 1.00E-137 | 95 |
| *YER182W* | *YER182W* | - | 96 | Q8N1Q8 | - | 4.00E-046 | 61 |
| *YER183C* | *FAU1* | - | 98 | P49914 | - | 1.00E-067 | 96 |
| *YFL001W* | *DEG1* | - | 97 | Q96J23 | - | 1.00E-111 | 81 |
| *YFL018C* | *LPD1* | yes | 98 | P09622 | yes | 1.00E-118 | 98 |
| *YFL036W* | *RPO41* | yes | 98 | O00411 | yes | 0 | 62 |
| *YFR019W* | *FAB1* | - | 93 | Q9Y2I7 | - | 0 | 97 |
| *YFR044C* | *YFR044C* | - | 97 | Q96KN2 | - | 0 | 98 |
| *YFR044C* | *YFR044C* | - | 97 | Q96KP4 | - | 0 | 99 |
| *YGL027C* | *CWH41* | - | 93 | Q13724 | - | 0 | 89 |
| *YGL055W* | *OLE1* | - | 93 | O00767 | - | 1.00E-123 | 77 |
| *YGL062W* | *PYC1* | - | 95 | P11498 | yes | 0 | 97 |
| *YGL068W* | *YGL068W* | - | 98 | P52815 | yes | 4.00E-041 | 70 |
| *YGL076C* | *RPL7A* | - | 95 | P18124 | - | 1.00E-099 | 100 |
| *YGL084C* | *GUP1* | - | 97 | Q9NVH9 | - | 1.00E-112 | 76 |
| *YGL103W* | *RPL28* | - | 95 | P46776 | - | 1.00E-066 | 100 |
| *YGL143C* | *MRF1* | yes | 98 | Q96EX4 | - | 2.00E-024 | 87 |
| *YGL187C* | *COX4* | yes | 98 | P10606 | yes | 9.00E-041 | 73 |
| *YGL191W* | *COX13* | yes | 98 | P12074 | yes | 3.00E-027 | 70 |
| *YGL211W* | *YGL211W* | - | 93 | Q96GZ7 | - | 2.00E-057 | 80 |
| *YGL236C* | *MTO1* | yes | 97 | Q8NDN7 | - | 0 | 95 |
| *YGL236C* | *MTO1* | yes | 97 | Q9Y2Z2 | - | 0 | 99 |
| *YGL240W* | *DOC1* | - | 92 | Q9UG51 | - | 4.00E-053 | 79 |
| *YGL256W* | *ADH4* | - | 95 | Q96MF9 | - | 1.00E-119 | 97 |
| *YGR020C* | *VMA7* | - | 96 | Q16864 | - | 2.00E-046 | 95 |
| *YGR021W* | *YGR021W* | - | 97 | Q9BSH4 | - | 2.00E-058 | 57 |
| *YGR082W* | *TOM20* | yes | 98 | Q15388 | yes | 1.00E-041 | 81 |
| *YGR094W* | *VAS1* | yes | 98 | Q9NSE4 | yes | 0 | 85 |
| *YGR094W* | *VAS1* | yes | 98 | P26640 | - | 0 | 89 |
| *YGR094W* | *VAS1* | yes | 98 | P41252 | - | 0 | 80 |
| *YGR094W* | *VAS1* | yes | 98 | Q96Q02 | - | 0 | 88 |
| *YGR094W* | *VAS1* | yes | 98 | Q9H6R2 | - | 0 | 98 |
| *YGR101W* | *PCP1* | yes | 97 | Q96CQ4 | - | 5.00E-057 | 58 |
| *YGR112W* | *SHY1* | yes | 98 | Q15526 | yes | 2.00E-079 | 76 |
| *YGR132C* | *PHB1* | yes | 98 | P35232 | - | 1.00E-097 | 96 |
| *YGR155W* | *CYS4* | - | 93 | P35520 | - | 1.00E-118 | 93 |
| *YGR183C* | *QCR9* | yes | 98 | Q9UDW1 | yes | 1.00E-017 | 80 |
| *YGR204W* | *ADE3* | - | 97 | P11586 | yes | 0 | 100 |
| *YGR204W* | *ADE3* | - | 97 | Q8WVW0 | - | 0 | 99 |
| *YGR204W* | *ADE3* | - | 97 | Q9UFU8 | - | 0 | 99 |
| *YGR207C* | *ETF-BETA* | yes | 97 | P38117 | yes | 1.00E-110 | 99 |
| *YGR214W* | *RPS0A* | - | 97 | P08865 | - | 1.00E-109 | 78 |
| *YGR220C* | *MRPL9* | yes | 98 | P09001 | yes | 1.00E-075 | 77 |
| *YGR240C* | *PFK1* | - | 96 | Q01813 | - | 0 | 95 |
| *YGR240C* | *PFK1* | - | 96 | Q9BR91 | - | 0 | 93 |
| *YGR244C* | *LSC2* | yes | 98 | Q9P2R7 | yes | 1.00E-173 | 92 |
| *YGR255C* | *COQ6* | yes | 98 | Q9Y2Z9 | - | 1.00E-115 | 96 |
| *YGR257C* | *YGR257C* | yes | 97 | Q9BZJ4 | yes | 2.00E-067 | 98 |
| *YHL021C* | *YHL021C* | - | 97 | Q9NVH6 | yes | 2.00E-096 | 80 |
| *YHL032C* | *GUT1* | - | 97 | P32189 | yes | 1.00E-175 | 98 |
| *YHL035C* | *YHL035C* | - | 93 | O15438 | - | 0 | 91 |
| *YHL035C* | *YHL035C* | - | 93 | P33527 | - | 0 | 91 |
| *YHL035C* | *YHL035C* | - | 93 | Q96J66 | - | 0 | 94 |
| *YHL035C* | *YHL035C* | - | 93 | Q9UQ97 | - | 0 | 91 |
| *YHR008C* | *SOD2* | yes | 98 | Q96EE6 | - | 2.00E-095 | 98 |
| *YHR011W* | *DIA4* | - | 97 | Q9NP81 | yes | 1.00E-117 | 93 |
| *YHR028C* | *DAP2* | - | 95 | P27487 | - | 0 | 96 |
| *YHR028C* | *DAP2* | - | 95 | P42658 | - | 0 | 89 |
| *YHR028C* | *DAP2* | - | 95 | Q12884 | - | 0 | 96 |
| *YHR028C* | *DAP2* | - | 95 | Q8N608 | - | 0 | 91 |
| *YHR038W* | *FIL1* | - | 98 | Q96E11 | - | 1.00E-052 | 77 |
| *YHR051W* | *COX6* | yes | 98 | P20674 | yes | 9.00E-060 | 97 |
| *YHR062C* | *RPP1* | yes | 96 | P78346 | - | 8.00E-084 | 82 |
| *YHR091C* | *MSR1* | yes | 98 | P54136 | - | 0 | 83 |
| *YHR091C* | *MSR1* | yes | 98 | Q96FU5 | - | 0 | 90 |
| *YHR120W* | *MSH1* | yes | 98 | P52701 | - | 0 | 100 |
| *YHR120W* | *MSH1* | yes | 98 | Q9BTB5 | - | 0 | 97 |
| *YHR203C* | *RPS4B* | - | 95 | P12750 | - | 1.00E-144 | 98 |
| *YHR208W* | *BAT1* | yes | 98 | Q96MY9 | - | 1.00E-151 | 94 |
| *YIL018W* | *RPL2B* | - | 96 | P25120 | - | 1.00E-104 | 100 |
| *YIL042C* | *YIL042C* | yes | 98 | Q15118 | yes | 1.00E-135 | 89 |
| *YIL051C* | *MMF1* | yes | 97 | P52758 | - | 1.00E-047 | 88 |
| *YIL065C* | *FIS1* | yes | 92 | Q9Y3D6 | - | 4.00E-034 | 62 |
| *YIL066C* | *RNR3* | - | 91 | P23921 | - | 0 | 96 |
| *YIL069C* | *RPS24B* | - | 95 | P16632 | - | 2.00E-040 | 98 |
| *YIL070C* | *MAM33* | yes | 98 | Q07021 | yes | 1.00E-058 | 97 |
| *YIL078W* | *THS1* | - | 91 | P26639 | - | 0 | 94 |
| *YIL078W* | *THS1* | - | 91 | Q9BW92 | - | 0 | 95 |
| *YIL093C* | *RSM25* | yes | 97 | Q9P053 | - | 5.00E-017 | 26 |
| *YIL124W* | *AYR1* | - | 94 | O14756 | - | 2.00E-050 | 95 |
| *YIL125W* | *KGD1* | yes | 98 | Q02218 | yes | 0 | 96 |
| *YIL125W* | *KGD1* | yes | 98 | Q96HY7 | - | 0 | 96 |
| *YIL125W* | *KGD1* | yes | 98 | Q9HCE2 | - | 0 | 94 |
| *YIL125W* | *KGD1* | yes | 98 | Q9NVA0 | - | 0 | 95 |
| *YIL125W* | *KGD1* | yes | 98 | Q9UDX0 | - | 0 | 96 |
| *YIL125W* | *KGD1* | yes | 98 | Q9ULD0 | - | 0 | 94 |
| *YIL133C* | *RPL16A* | - | 95 | P40429 | - | 1.00E-073 | 95 |
| *YIL155C* | *GUT2* | yes | 98 | P43304 | yes | 0 | 85 |
| *YJL003W* | *COX16* | yes | 97 | Q9P0S2 | - | 2.00E-025 | 73 |
| *YJL045W* | *YJL045W* | yes | 98 | P31040 | yes | 0 | 100 |
| *YJL046W* | *YJL046W* | - | 97 | Q9Y234 | yes | 1.00E-117 | 68 |
| *YJL060W* | *BNA3* | - | 96 | Q16773 | - | 3.00E-079 | 93 |
| *YJL063C* | *MRPL8* | yes | 98 | Q9NRX2 | yes | 5.00E-045 | 64 |
| *YJL088W* | *ARG3* | - | 95 | P00480 | yes | 1.00E-122 | 90 |
| *YJL104W* | *MIA1* | - | 98 | Q9Y3D7 | yes | 2.00E-040 | 99 |
| *YJL130C* | *URA2* | - | 97 | P31327 | yes | 0 | 96 |
| *YJL130C* | *URA2* | - | 97 | P27708 | - | 0 | 100 |
| *YJL143W* | *TIM17* | yes | 96 | O60830 | yes | 2.00E-049 | 96 |
| *YJL177W* | *RPL17B* | - | 95 | P18621 | - | 2.00E-090 | 80 |
| *YJL180C* | *ATP12* | yes | 98 | Q8N5M1 | - | 7.00E-077 | 78 |
| *YJL200C* | *YJL200C* | - | 97 | Q99798 | yes | 0 | 96 |
| *YJL200C* | *YJL200C* | - | 97 | O75944 | - | 0 | 100 |
| *YJL200C* | *YJL200C* | - | 97 | P21399 | - | 0 | 98 |
| *YJL208C* | *NUC1* | yes | 97 | Q9Y2C4 | - | 3.00E-097 | 78 |
| *YJR048W* | *CYC1* | yes | 98 | P00001 | yes | 5.00E-043 | 97 |
| *YJR100C* | *YJR100C* | - | 91 | O15162 | - | 3.00E-084 | 76 |
| *YJR104C* | *SOD1* | yes | 97 | P00441 | - | 7.00E-058 | 98 |
| *YJR109C* | *CPA2* | - | 97 | P31327 | yes | 0 | 95 |
| *YJR117W* | *STE24* | - | 95 | O75844 | - | 1.00E-138 | 98 |
| *YJR121W* | *ATP2* | yes | 98 | P06576 | yes | 0 | 97 |
| *YJR145C* | *RPS4A* | - | 95 | P12750 | - | 1.00E-144 | 98 |
| *YKL003C* | *MRP17* | yes | 98 | P82932 | yes | 1.00E-034 | 72 |
| *YKL016C* | *ATP7* | yes | 98 | O75947 | yes | 4.00E-050 | 89 |
| *YKL029C* | *MAE1* | yes | 98 | P23368 | yes | 0 | 96 |
| *YKL029C* | *MAE1* | yes | 98 | Q16798 | yes | 0 | 93 |
| *YKL029C* | *MAE1* | yes | 98 | P48163 | - | 0 | 96 |
| *YKL040C* | *NFU1* | yes | 98 | Q9UMS0 | - | 5.00E-082 | 96 |
| *YKL134C* | *10/01/04* | yes | 98 | Q96G65 | yes | 0 | 88 |
| *YKL134C* | *10/01/04* | yes | 98 | Q99797 | yes | 0 | 88 |
| *YKL134C* | *10/01/04* | yes | 98 | P52888 | - | 0 | 92 |
| *YKL141W* | *SDH3* | yes | 98 | Q99643 | yes | 4.00E-054 | 82 |
| *YKL148C* | *SDH1* | yes | 98 | P31040 | yes | 0 | 97 |
| *YKL192C* | *ACP1* | yes | 98 | O14561 | yes | 1.00E-034 | 94 |
| *YKL212W* | *SAC1* | - | 95 | O94935 | - | 0 | 96 |
| *YKR009C* | *FOX2* | - | 91 | P51659 | - | 3.00E-055 | 38 |
| *YKR070W* | *YKR070W* | - | 97 | Q9BXW7 | - | 1.00E-086 | 98 |
| *YLL001W* | *DNM1* | yes | 96 | O00429 | - | 0 | 99 |
| *YLL001W* | *DNM1* | yes | 96 | P50570 | - | 0 | 97 |
| *YLL001W* | *DNM1* | yes | 96 | Q8TBT7 | - | 0 | 99 |
| *YLL001W* | *DNM1* | yes | 96 | Q9UQ16 | - | 0 | 96 |
| *YLL027W* | *ISA1* | yes | 97 | Q9BZR2 | - | 7.00E-041 | 82 |
| *YLL040C* | *VPS13* | - | 91 | Q96RL7 | - | 0 | 100 |
| *YLL040C* | *VPS13* | - | 91 | Q9P2C6 | - | 0 | 94 |
| *YLL041C* | *SDH2* | yes | 98 | P21912 | yes | 1.00E-131 | 94 |
| *YLR038C* | *COX12* | yes | 98 | P14854 | yes | 9.00E-026 | 88 |
| *YLR056W* | *ERG3* | - | 96 | O75845 | - | 7.00E-083 | 95 |
| *YLR058C* | *SHM2* | - | 91 | P34896 | yes | 0 | 99 |
| *YLR058C* | *SHM2* | - | 91 | P34897 | yes | 0 | 96 |
| *YLR059C* | *REX2* | yes | 98 | Q9Y3B8 | yes | 2.00E-083 | 74 |
| *YLR067C* | *PET309* | yes | 96 | P42704 | - | 3.00E-023 | 89 |
| *YLR075W* | *RPL10* | - | 95 | P27635 | - | 1.00E-109 | 97 |
| *YLR089C* | *YLR089C* | - | 97 | Q8TD30 | - | 1.00E-107 | 94 |
| *YLR106C* | *MDN1* | - | 96 | Q9NU22 | - | 0 | 24 |
| *YLR142W* | *PUT1* | yes | 98 | Q9UF12 | - | 1.00E-133 | 74 |
| *YLR163C* | *MAS1* | yes | 98 | O75439 | yes | 1.00E-162 | 96 |
| *YLR201C* | *YLR201C* | - | 98 | Q9P056 | - | 1.00E-074 | 75 |
| *YLR218C* | *YLR218C* | - | 96 | Q9NYJ1 | - | 8.00E-022 | 59 |
| *YLR239C* | *LIP2* | yes | 98 | O75627 | - | 3.00E-036 | 98 |
| *YLR244C* | *MAP1* | - | 93 | P53582 | - | 1.00E-114 | 94 |
| *YLR259C* | *HSP60* | yes | 98 | P10809 | yes | 1.00E-166 | 95 |
| *YLR270W* | *DCS1* | - | 93 | Q96C86 | - | 1.00E-111 | 84 |
| *YLR270W* | *DCS1* | - | 93 | Q9Y2S5 | - | 1.00E-111 | 84 |
| *YLR289W* | *GUF1* | - | 93 | Q9H8U4 | - | 1.00E-147 | 97 |
| *YLR304C* | *ACO1* | yes | 98 | Q99798 | yes | 0 | 98 |
| *YLR304C* | *ACO1* | yes | 98 | O75944 | - | 0 | 99 |
| *YLR304C* | *ACO1* | yes | 98 | P21399 | - | 0 | 100 |
| *YLR305C* | *STT4* | - | 96 | P42356 | - | 0 | 100 |
| *YLR305C* | *STT4* | - | 96 | Q9UPG2 | - | 0 | 78 |
| *YLR340W* | *RPP0* | - | 97 | P05388 | - | 1.00E-136 | 98 |
| *YLR382C* | *NAM2* | yes | 98 | Q15031 | yes | 0 | 94 |
| *YLR439W* | *MRPL4* | yes | 98 | Q8N5D1 | yes | 3.00E-049 | 59 |
| *YML021C* | *UNG1* | yes | 97 | P13051 | yes | 1.00E-115 | 71 |
| *YML025C* | *YML6* | yes | 98 | Q9BYD3 | yes | 2.00E-074 | 65 |
| *YML030W* | *YML030W* | - | 96 | Q9BW72 | - | 2.00E-025 | 60 |
| *YML042W* | *CAT2* | yes | 97 | P43155 | yes | 0 | 95 |
| *YML042W* | *CAT2* | yes | 97 | P50416 | yes | 0 | 96 |
| *YML042W* | *CAT2* | yes | 97 | Q92523 | yes | 0 | 95 |
| *YML042W* | *CAT2* | yes | 97 | P28329 | - | 0 | 89 |
| *YML042W* | *CAT2* | yes | 97 | Q8TCG5 | - | 0 | 95 |
| *YML042W* | *CAT2* | yes | 97 | Q9UKG9 | - | 0 | 98 |
| *YML054C* | *CYB2* | yes | 97 | Q9UJM8 | - | 1.00E-121 | 97 |
| *YML110C* | *COQ5* | yes | 98 | Q9BSP8 | - | 1.00E-048 | 97 |
| *YML126C* | *ERG13* | - | 91 | P54868 | yes | 0 | 94 |
| *YML126C* | *ERG13* | - | 91 | Q01581 | - | 0 | 93 |
| *YML126C* | *ERG13* | - | 91 | Q8N7N8 | - | 0 | 93 |
| *YMR012W* | *CLU1* | - | 94 | O75153 | - | 1.00E-090 | 89 |
| *YMR023C* | *MSS1* | yes | 98 | Q9BX61 | yes | 1.00E-060 | 97 |
| *YMR035W* | *IMP2* | yes | 98 | Q96T52 | yes | 2.00E-049 | 82 |
| *YMR060C* | *TOM37* | yes | 98 | Q13505 | yes | 5.00E-063 | 57 |
| *YMR125W* | *STO1* | - | 92 | Q09161 | - | 1.00E-121 | 40 |
| *YMR167W* | *MLH1* | - | 95 | P40692 | - | 0 | 99 |
| *YMR186W* | *HSC82* | - | 96 | O75322 | - | 0 | 76 |
| *YMR186W* | *HSC82* | - | 96 | P14625 | - | 0 | 96 |
| *YMR189W* | *GCV2* | yes | 97 | P23378 | yes | 0 | 93 |
| *YMR203W* | *TOM40* | yes | 94 | Q969M1 | - | 1.00E-105 | 93 |
| *YMR207C* | *HFA1* | - | 98 | P05165 | yes | 0 | 93 |
| *YMR207C* | *HFA1* | - | 98 | Q96RQ3 | yes | 0 | 92 |
| *YMR207C* | *HFA1* | - | 98 | O00763 | - | 0 | 100 |
| *YMR207C* | *HFA1* | - | 98 | Q13085 | - | 0 | 99 |
| *YMR242C* | *RPL20A* | - | 95 | Q02543 | - | 2.00E-075 | 96 |
| *YMR267W* | *PPA2* | yes | 98 | Q9H2U2 | - | 1.00E-147 | 98 |
| *YMR290C* | *HAS1* | - | 95 | Q9NVP1 | - | 1.00E-105 | 99 |
| *YMR293C* | *YMR293C* | yes | 98 | Q9H0R6 | - | 1.00E-139 | 95 |
| *YNL005C* | *MRP7* | yes | 98 | Q9P0M9 | yes | 3.00E-036 | 56 |
| *YNL026W* | *YNL026W* | - | 97 | Q9Y512 | - | 2.00E-091 | 52 |
| *YNL037C* | *IDH1* | yes | 98 | P51553 | yes | 1.00E-139 | 95 |
| *YNL055C* | *POR1* | yes | 98 | P21796 | yes | 1.00E-107 | 99 |
| *YNL064C* | *YDJ1* | - | 93 | P31689 | yes | 1.00E-135 | 99 |
| *YNL067W* | *RPL9B* | - | 96 | P32969 | - | 7.00E-086 | 97 |
| *YNL088W* | *TOP2* | - | 97 | P11388 | - | 0 | 88 |
| *YNL088W* | *TOP2* | - | 97 | Q02880 | - | 0 | 91 |
| *YNL168C* | *YNL168C* | - | 97 | Q9H0N6 | - | 9.00E-083 | 94 |
| *YNL185C* | *MRPL19* | yes | 98 | Q9Y3B7 | yes | 2.00E-050 | 87 |
| *YNL200C* | *YNL200C* | - | 96 | Q8NCW5 | - | 1.00E-102 | 94 |
| *YNL239W* | *LAP3* | - | 93 | Q13867 | - | 0 | 100 |
| *YNL252C* | *MRPL17* | yes | 98 | Q9H2W6 | yes | 2.00E-056 | 52 |
| *YNL284C* | *MRPL10* | yes | 98 | Q9P015 | - | 2.00E-058 | 67 |
| *YNL315C* | *ATP11* | yes | 98 | Q9H6E3 | - | 2.00E-067 | 54 |
| *YNR001C* | *CIT1* | yes | 98 | O75390 | yes | 0 | 96 |
| *YNR016C* | *ACC1* | - | 97 | P05165 | yes | 0 | 95 |
| *YNR016C* | *ACC1* | - | 97 | Q96RQ3 | yes | 0 | 93 |
| *YNR016C* | *ACC1* | - | 97 | O00763 | - | 0 | 98 |
| *YNR016C* | *ACC1* | - | 97 | Q13085 | - | 0 | 98 |
| *YNR017W* | *MAS6* | yes | 97 | O14925 | yes | 4.00E-048 | 88 |
| *YNR036C* | *YNR036C* | - | 97 | O15235 | yes | 3.00E-041 | 81 |
| *YNR041C* | *COQ2* | yes | 98 | Q96H96 | - | 1.00E-108 | 78 |
| *YOL008W* | *YOL008W* | - | 95 | Q8TAL2 | - | 2.00E-069 | 85 |
| *YOL021C* | *DIS3* | - | 96 | Q8N1N8 | - | 0 | 97 |
| *YOL021C* | *DIS3* | - | 96 | Q8WTU9 | - | 0 | 90 |
| *YOL021C* | *DIS3* | - | 96 | Q9Y2L1 | - | 0 | 94 |
| *YOL023W* | *IFM1* | yes | 98 | P46199 | yes | 1.00E-125 | 97 |
| *YOL027C* | *MDM38* | - | 98 | O95202 | - | 1.00E-132 | 57 |
| *YOL033W* | *MSE1* | yes | 98 | Q8TF31 | - | 1.00E-178 | 94 |
| *YOL040C* | *RPS15* | - | 97 | P11174 | - | 1.00E-060 | 99 |
| *YOL071W* | *EMI5* | - | 97 | Q9NX18 | - | 5.00E-041 | 78 |
| *YOL120C* | *RPL18A* | - | 96 | Q07020 | - | 1.00E-068 | 83 |
| *YOR020C* | *HSP10* | yes | 98 | Q04984 | yes | 4.00E-028 | 92 |
| *YOR063W* | *RPL3* | - | 94 | P39023 | - | 0 | 100 |
| *YOR065W* | *CYT1* | yes | 98 | P08574 | yes | 1.00E-127 | 79 |
| *YOR090C* | *PTC5* | - | 92 | Q9P2J9 | yes | 2.00E-096 | 90 |
| *YOR108W* | *LEU9* | - | 97 | P35914 | yes | 8.00E-097 | 90 |
| *YOR125C* | *CAT5* | yes | 97 | Q99807 | yes | 2.00E-084 | 81 |
| *YOR133W* | *EFT1* | - | 91 | P13639 | - | 0 | 99 |
| *YOR133W* | *EFT1* | - | 91 | Q15029 | - | 0 | 100 |
| *YOR142W* | *LSC1* | yes | 98 | P53597 | yes | 1.00E-120 | 98 |
| *YOR150W* | *MRPL23* | yes | 98 | Q9BYD1 | yes | 8.00E-052 | 88 |
| *YOR151C* | *RPB2* | - | 91 | P30876 | - | 0 | 99 |
| *YOR176W* | *HEM15* | yes | 98 | P22830 | yes | 1.00E-164 | 88 |
| *YOR196C* | *LIP5* | yes | 98 | O43766 | yes | 2.00E-053 | 97 |
| *YOR241W* | *MET7* | yes | 96 | Q05932 | yes | 1.00E-171 | 81 |
| *YOR251C* | *YOR251C* | - | 93 | P25325 | yes | 1.00E-109 | 96 |
| *YOR271C* | *YOR271C* | - | 94 | Q9H9B4 | yes | 1.00E-131 | 100 |
| *YOR274W* | *MOD5* | yes | 97 | Q9H3H1 | - | 1.00E-151 | 96 |
| *YOR278W* | *HEM4* | - | 92 | P10746 | yes | 4.00E-058 | 61 |
| *YOR286W* | *YOR286W* | - | 96 | Q8NFU3 | - | 4.00E-025 | 99 |
| *YOR317W* | *FAA1* | - | 97 | P33121 | yes | 1.00E-177 | 94 |
| *YOR330C* | *MIP1* | yes | 98 | P54098 | yes | 0 | 94 |
| *YOR334W* | *MRS2* | yes | 98 | Q9HD23 | yes | 1.00E-110 | 81 |
| *YOR335C* | *ALA1* | - | 95 | P49588 | - | 0 | 99 |
| *YOR335C* | *ALA1* | - | 95 | Q9ULF0 | - | 0 | 93 |
| *YOR356W* | *YOR356W* | yes | 97 | Q16134 | yes | 0 | 89 |
| *YOR374W* | *ALD4* | yes | 97 | P47895 | - | 0 | 96 |
| *YOR374W* | *ALD4* | yes | 97 | Q8NHQ4 | - | 0 | 94 |
| *YOR386W* | *PHR1* | - | 94 | Q16526 | yes | 1.00E-159 | 86 |
| *YPL013C* | *MRPS16* | yes | 98 | Q9Y3D3 | yes | 2.00E-035 | 66 |
| *YPL028W* | *ERG10* | - | 96 | P24752 | yes | 1.00E-145 | 97 |
| *YPL028W* | *ERG10* | - | 96 | Q8TDM4 | - | 1.00E-145 | 99 |
| *YPL040C* | *ISM1* | yes | 98 | Q9NSE4 | yes | 0 | 98 |
| *YPL091W* | *GLR1* | - | 97 | P00390 | yes | 1.00E-120 | 99 |
| *YPL097W* | *MSY1* | yes | 98 | Q9H817 | - | 0 | 92 |
| *YPL104W* | *MSD1* | yes | 98 | Q9H455 | - | 1.00E-173 | 92 |
| *YPL131W* | *RPL5* | - | 94 | Q9H3F4 | - | 1.00E-120 | 85 |
| *YPL132W* | *COX11* | yes | 98 | Q9Y6N1 | yes | 2.00E-092 | 67 |
| *YPL135W* | *ISU1* | yes | 97 | Q9H1K1 | - | 1.00E-064 | 80 |
| *YPL172C* | *COX10* | yes | 98 | Q12887 | yes | 1.00E-138 | 86 |
| *YPL183W-A* | *YPL183W-A* | yes | 97 | Q9P0J6 | yes | 5.00E-018 | 95 |
| *YPL206C* | *YPL206C* | - | 96 | Q9NZC3 | - | 9.00E-059 | 80 |
| *YPL215W* | *CBP3* | yes | 98 | Q9NVA1 | - | 3.00E-066 | 48 |
| *YPL240C* | *HSP82* | - | 91 | O75322 | - | 0 | 76 |
| *YPL252C* | *YAH1* | yes | 98 | P10109 | yes | 2.00E-042 | 66 |
| *YPL262W* | *FUM1* | yes | 98 | P07954 | yes | 0 | 98 |
| *YPR004C* | *YPR004C* | - | 97 | P13804 | yes | 1.00E-142 | 85 |
| *YPR021C* | *YPR021C* | yes | 98 | Q9UJS0 | yes | 1.00E-046 | 39 |
| *YPR033C* | *HTS1* | yes | 97 | P12081 | - | 1.00E-180 | 89 |
| *YPR047W* | *MSF1* | yes | 98 | O95363 | yes | 1.00E-166 | 89 |
| *YPR100W* | *MRPL51* | yes | 97 | Q8N983 | - | 2.00E-041 | 85 |
| *YPR140W* | *YPR140W* | - | 96 | Q16635 | yes | 1.00E-094 | 87 |
| *YPR166C* | *MRP2* | yes | 98 | O60783 | yes | 1.00E-030 | 86 |
| *YPR183W* | *DPM1* | - | 97 | Q9H9J8 | - | 4.00E-069 | 93 |

The table contains human orthologous proteins (reciprocal best hits) identified for 399 of 477 mitochondrial reference proteins and for 292 new candidates, all of which have MitoP2 scores >90 as calculated by integrative analysis.

^a^‘yes’ indicates that the protein is among the 477 known mitochondrial proteins in yeast.

^b^The homology search using PSI-BLAST algorithm identified 370 unique human proteins, of which 151 are annotated as human mitochondrial proteins in MitoP2.

^c^The BLAST e-values of the match, and ^d^the coverage of the BLAST alignment defined as the percent of amino acids of the shorter protein covered by the alignment.
