# Supplementary material for: Integrative Analysis of the Mitochondrial Proteome in Yeast
Source: PLoS Biol. 2004 Jun 15;2(6):e160. doi: 10.1371/journal.pbio.0020160 (PMC423137; doi:10.1371/journal.pbio.0020160)
Supplement: Table S4 — (224 KB DOC). [file pbio.0020160.st004.doc]

**Table S3.** Members of selected mitochondrial protein complexes.

| Yeast  ORF | Gene | Mitochondrial  localization^a^ | Proteome ranking^b^ | MitoP2  score | Protein  complex^c^ | NF  max^d^ | F  max^e^ |
| --- | --- | --- | --- | --- | --- | --- | --- |
| *YAL044C* | *GCV3* | MATRIX | H_115 | 98 | GlycineDC | 8 | 8 |
| *YDR019C* | *GCV1* | MATRIX | H_078 | 98 | GlycineDC | 12 | 11 |
| *YMR189W* | *GCV2* | MATRIX | H_122 | 97 | GlycineDC | 22 | 31 |
| *YBR221C* | *PDB1* | MATRIX | H_065 | 98 | PDH | 18 | 20 |
| *YER178W* | *PDA1* | MATRIX | H_061 | 98 | PDH | 14 | 16 |
| *YGR193C* | *PDX1* | MATRIX | H_151 | 98 | PDH | 10 | 11 |
| *YNL071W* | *LAT1* | MATRIX | H_045 | 98 | PDH | 21 | 27 |
| *YER017C* | *AFG3* | MIM | M_026 | 98 | PROTEASE | 4 | 3 |
| *YGR132C* | *PHB1* | MIM | H_064 | 98 | PROTEASE | 9 | 10 |
| *YGR231C* | *PHB2* | MIM | H_081 | 97 | PROTEASE | 6 | 9 |
| *YMR089C* | *YTA12* | MIM | M_007 | 98 | PROTEASE | 4 | 1 |
| *YPR024W* | *YME1* | MIM | H_182 | 98 | PROTEASE (I-AAA) | 4 | 6 |
| *YDL085W* |  | MIM | M_027 | 95 | RCC1 | 5 | 0 |
| *YML120C* | *NDI1* | MIM | H_050 | 98 | RCC1 | 24 | 23 |
| *YMR145C* |  | MIM | H_051 | 98 | RCC1 | 18 | 18 |
| *YDR178W* | *SDH4* | MIM | H_107 | 98 | RCC2 | 3 | 2 |
| *YKL141W* | *SDH3* | MIM | H_074 | 98 | RCC2 | 3 | 3 |
| *YKL148C* | *SDH1* | MIM | H_007 | 98 | RCC2 | 17 | 14 |
| *YLL041C* | *SDH2* | MIM | H_056 | 98 | RCC2 | 12 | 6 |
| *YBR044C* | *TCM62* | MIM | - | 98 | RCC2_Asy | 1 | 1 |
| *Q0105* | *COB* | MIM | - | 97 | RCC3 | 1 | 0 |
| *YBL045C* | *COR1* | MIM | H_006 | 98 | RCC3 | 29 | 27 |
| *YDR529C* | *QCR7* | MIM | H_019 | 98 | RCC3 | 10 | 10 |
| *YEL024W* | *RIP1* | MIM | H_028 | 98 | RCC3 | 12 | 9 |
| *YFR033C* | *QCR6* | MIM | M_158 | 98 | RCC3 | 2 | 1 |
| *YGR183C* | *QCR9* | MIM | - | 98 | RCC3 | 1 | 0 |
| *YHR001W-A* | *QCR10* | MIM | H_130 | 89 | RCC3 | 4 | 2 |
| *YJL166W* | *QCR8* | MIM | H_132 | 98 | RCC3 | 4 | 2 |
| *YOR065W* | *CYT1* | MIM | H_069 | 98 | RCC3 | 8 | 7 |
| *YPR191W* | *QCR2* | MIM | H_005 | 98 | RCC3 | 32 | 29 |
| *YPL215W* | *CBP3* | MITO | H_159 | 98 | RCC3_Asy | 7 | 6 |
| *Q0045* | COX1 | MIM | H_133 | 97 | RCC4 | 3 | 3 |
| *Q0250* | *COX2* | MIM | H_040 | 97 | RCC4 | 8 | 5 |
| *Q0275* | *COX3* | MIM | - | 97 | RCC4 | 0 | 0 |
| *YDL067C* | *COX9* | MIM | H_120 | 97 | RCC4 | 2 | 2 |
| *YGL187C* | *COX4* | MIM | H_018 | 98 | RCC4 | 9 | 8 |
| *YGL191W* | *COX13* | MIM | H_083 | 98 | RCC4 | 10 | 6 |
| *YHR051W* | *COX6* | MIM | H_013 | 98 | RCC4 | 10 | 12 |
| *YIL111W* | *COX5B* | MIM | H_136 | 98 | RCC4 | 2 | 3 |
| *YLR038C* | *COX12* | MIM | H_108 | 98 | RCC4 | 3 | 1 |
| *YLR395C* | *COX8* | MIM | M_018 | 98 | RCC4 | 2 | 1 |
| *YMR256C* | *COX7* | MIM | H_171 | 97 | RCC4 | 3 | 2 |
| *YNL052W* | *COX5A* | MIM | H_055 | 98 | RCC4 | 9 | 9 |
| *YBR024W* | *SCO2* | MIM | - | 98 | RCC4_Asy | 1 | 1 |
| *YBR037C* | *SCO1* | MIM | M_103 | 98 | RCC4_Asy | 2 | 2 |
| *YBR185C* | *MBA1* | MIM | - | 98 | RCC4_Asy | 1 | 0 |
| *YDR079W* | *PET100* | MIM | - | 98 | RCC4_Asy | 0 | 0 |
| *YDR231C* | *COX20* | MIM | M_209 | 97 | RCC4_Asy | 2 | 0 |
| *YER058W* | *PET117* | MIM | - | 98 | RCC4_Asy | 1 | 1 |
| *YER141W* | *COX15* | MIM | H_145 | 98 | RCC4_Asy | 4 | 4 |
| *YGR062C* | *COX18* | MIM | - | 97 | RCC4_Asy | 0 | 0 |
| *YGR112W* | *SHY1* | MIM | M_132 | 98 | RCC4_Asy | 4 | 0 |
| *YJL003W* |  | MIM | - | 97 | RCC4_Asy | 1 | 0 |
| *YJR034W* | *PET191* | MIM | - | 98 | RCC4_Asy | 0 | 0 |
| *YLL009C* | *COX17* | MIMS | - | 98 | RCC4_Asy | 0 | 0 |
| *YLL018C-A* | *COX19* | MIMS | - | 92 | RCC4_Asy | 0 | 0 |
| *YML129C* | *COX14* | MIM | - | 98 | RCC4_Asy | 1 | 1 |
| *YPL132W* | *COX11* | MIM | M_102 | 98 | RCC4_Asy | 3 | 1 |
| *YPL172C* | *COX10* | MIM | - | 98 | RCC4_Asy | 1 | 0 |
| *Q0080* | *AAP1* | MIM | - | 51 | RCC5 | 1 | 1 |
| *Q0085* | *ATP6* | MIM | H_208 | 97 | RCC5 | 2 | 1 |
| *Q0130* | *OLI1* | MIM | - | 97 | RCC5 | 0 | 0 |
| *YBL099W* | *ATP1* | MIM | H_002 | 98 | RCC5 | 50 | 49 |
| *YBR039W* | *ATP3* | MIM | H_011 | 98 | RCC5 | 22 | 20 |
| *YDL004W* | *ATP16* | MIM | H_070 | 98 | RCC5 | 9 | 9 |
| *YDL130W-A* | *STF1* | MIM | H_157 | 93 | RCC5 | 7 | 3 |
| *YDL181W* | *INH1* | MIM | H_124 | 98 | RCC5 | 8 | 4 |
| *YDR298C* | *ATP5* | MIM | H_012 | 98 | RCC5 | 13 | 10 |
| *YDR322C-A* | *TIM11* | MIM | H_073 | 89 | RCC5 | 8 | 7 |
| *YDR377W* | *ATP17* | MIM | H_117 | 98 | RCC5 | 6 | 6 |
| *YGR008C* | *STF2* | MIM | - | 18 | RCC5 | 0 | 0 |
| *YJR121W* | *ATP2* | MIM | H_008 | 98 | RCC5 | 61 | 74 |
| *YKL016C* | *ATP7* | MIM | H_017 | 98 | RCC5 | 13 | 13 |
| *YLR295C* | *ATP14* | MIM | H_087 | 98 | RCC5 | 5 | 5 |
| *YML081C-A* | *ATP18* | MIM | H_109 | 98 | RCC5 | 3 | 1 |
| *YOL077W-A* | *ATP19* | MIM | H_162 | 81 | RCC5 | 4 | 2 |
| *YPL078C* | *ATP4* | MIM | H_026 | 98 | RCC5 | 17 | 17 |
| *YPL271W* | *ATP15* | MIM | H_071 | 98 | RCC5 | 6 | 5 |
| *YPR020W* | *ATP20* | MIM | H_135 | 97 | RCC5 | 3 | 3 |
| *YIL098C* | *FMC1* | MATRIX | M_215 | 98 | RCC5_Asy | 2 | 1 |
| *YJL180C* | *ATP12* | MATRIX | - | 98 | RCC5_Asy | 1 | 1 |
| *YLR393W* | *ATP10* | MIM | M_147 | 98 | RCC5_Asy | 2 | 1 |
| *YNL315C* | *ATP11* | MATRIX | M_201 | 98 | RCC5_Asy | 2 | 1 |
| *YDR148C* | *KGD2* | MATRIX | H_027 | 98 | TCA | 18 | 12 |
| *YFL018C* | *LPD1* | MATRIX | H_021 | 98 | TCA | 31 | 27 |
| *YGR244C* | *LSC2* | MATRIX | H_024 | 98 | TCA | 19 | 19 |
| *YIL125W* | *KGD1* | MATRIX | H_015 | 98 | TCA | 44 | 30 |
| *YKL085W* | *MDH1* | MATRIX | H_020 | 98 | TCA | 34 | 31 |
| *YLR304C* | *ACO1* | MATRIX | H_001 | 98 | TCA | 67 | 47 |
| *YNL037C* | *IDH1* | MATRIX | H_010 | 98 | TCA | 28 | 29 |
| *YNR001C* | *CIT1* | MATRIX | H_004 | 98 | TCA | 54 | 47 |
| *YOR136W* | *IDH2* | MATRIX | H_035 | 98 | TCA | 19 | 17 |
| *YOR142W* | *LSC1* | MATRIX | H_034 | 98 | TCA | 17 | 16 |
| *YPL262W* | *FUM1* | MATRIX | H_037 | 98 | TCA | 21 | 14 |
| *YPR001W* | *CIT3* | MATRIX | H_155 | 98 | TCA | 8 | 3 |
| *YBR091C* | *MRS5* | MIMS | - | 97 | TIM22/8/13 | 0 | 0 |
| *YDL217C* | *TIM22* | MIM | - | 93 | TIM22/8/13 | 0 | 0 |
| *YEL020W-A* | *TIM9* | MIMS | M_064 | 94 | TIM22/8/13 | 3 | 0 |
| *YGR181W* | *TIM13* | MIMS | - | 87 | TIM22/8/13 | 1 | 0 |
| *YHR005C-A* | *MRS11* | MIMS | H_174 | 49 | TIM22/8/13 | 3 | 1 |
| *YJL054W* | *TIM54* | MIM | M_077 | 97 | TIM22/8/13 | 2 | 1 |
| *YJR135W-A* | *TIM8* | MIMS | - | 80 | TIM22/8/13 | 1 | 0 |
| *YOR297C* | *TIM18* | MIM | - | 97 | TIM22/8/13 | 1 | 0 |
| *YFL016C* | *MDJ1* | MIM | H_141 | 98 | TIM23 | 10 | 11 |
| *YIL022W* | *TIM44* | MATRIX | H_125 | 97 | TIM23 | 8 | 7 |
| *YJL143W* | *TIM17* | MIM | - | 96 | TIM23 | 0 | 0 |
| *YJR045C* | *SSC1* | MATRIX | H_009 | 98 | TIM23 | 57 | 55 |
| *YNR017W* | *MAS6* | MIM | H_183 | 97 | TIM23 | 5 | 5 |
| *YOR232W* | *MGE1* | MATRIX | H_102 | 98 | TIM23 | 8 | 10 |
| *YPL063W* |  | MIM | H_181 | 89 | TIM23 | 4 | 6 |
| *YGR082W* | *TOM20* | MOM | H_206 | 98 | TOM | 3 | 2 |
| *YHR117W* | *TOM71* | MOM | M_039 | 90 | TOM | 3 | 2 |
| *YMR060C* | *TOM37* | MOM | - | 98 | TOM | 1 | 1 |
| *YMR203W* | *TOM40* | MOM | H_032 | 94 | TOM | 7 | 6 |
| *YNL070W* | *TOM7* | MOM | M_017 | 97 | TOM | 2 | 2 |
| *YNL121C* | *TOM70* | MOM | H_039 | 97 | TOM | 16 | 13 |
| *YNL131W* | *TOM22* | MOM | H_105 | 93 | TOM | 2 | 5 |
| *YOR045W* | *TOM6* | MOM | - | 91 | TOM | 0 | 0 |
| *YPR133W-A* | *TOM5* | MOM | H_147 | 97 | TOM | 2 | 1 |

^a^Mitochondrial localization: MITO, mitochondrial with unspecified sub-localization; MOM, mitochondrial outer membrane; MIMS, mitochondrial intermembrane space; MIM, mitochondrial inner membrane; MATRIX, mitochondrial matrix.

^b^Proteome Ranking: H, High confidence class in proteomics; M, Medium confidence class; L, Low confidence class; the number indicates the ranking position within each confidence class according to number of experiments in which the protein was detected; dash by itself means not detected.

^c^Protein complex: GlycineDC, Glycine decarboxylase; NDH, NADH-oxidoreductase; PDH, Pyruvate dehydrogenase; PROTEASE, mitochondrial protease complex; RCC, mitochondrial respiratory chain complex (1-5); Asy, assembly factors of the respiratory chain complex; TIM22/8/13 and TIM23, transport across inner membrane; TOM, transport across outer membrane.

^d^NF max: maximum number of tags detected under non-fermentable growth conditions. ^e^F max: maximum tag number detected under fermentable media conditions.
